# Imaging of intestinal vasculitis focusing on MR and CT enterography: a two-way street between radiologic findings and clinical data

**DOI:** 10.1186/s13244-022-01284-7

**Published:** 2022-09-04

**Authors:** Mehrnam Amouei, Sara Momtazmanesh, Hoda Kavosi, Amir H. Davarpanah, Ali Shirkhoda, Amir Reza Radmard

**Affiliations:** 1 Department of Radiology, Shariati Hospital, Tehran University of Medical Sciences, North Kargar St., Tehran, 14117 Iran; 2 Department of Rheumatology, Shariati Hospital, Tehran University of Medical Sciences, Tehran, Iran; 3 Department of Radiology and Imaging Sciences, Emory University School of Medicine, Atlanta, USA; 4 Department of Radiological Science, University of California at Irvine, Irvine, USA

**Keywords:** Magnetic resonance enterography, Computed tomography enterography, Vasculitis, Intestines

## Abstract

Diagnosis of intestinal vasculitis is often challenging due to the non-specific clinical and imaging findings. Vasculitides with gastrointestinal (GI) manifestations are rare, but their diagnosis holds immense significance as late or missed recognition can result in high mortality rates. Given the resemblance of radiologic findings with some other entities, GI vasculitis is often overlooked on small bowel studies done using computed tomography/magnetic resonance enterography (CTE/MRE). Hereon, we reviewed radiologic findings of vasculitis with gastrointestinal involvement on CTE and MRE. The variety of findings on MRE/CTE depend upon the size of the involved vessels. Signs of intestinal ischemia, e.g., mural thickening, submucosal edema, mural hyperenhancement, and restricted diffusion on diffusion-weighted imaging, are common in intestinal vasculitis. Involvement of the abdominal aorta and the major visceral arteries is presented as concentric mural thickening, transmural calcification, luminal stenosis, occlusion, aneurysmal changes, and collateral vessels. Such findings can be observed particularly in large- and medium-vessel vasculitis. The presence of extra-intestinal findings, including within the liver, kidneys, or spleen in the form of focal areas of infarction or heterogeneous enhancement due to microvascular involvement, can be another radiologic clue in diagnosis of vasculitis**.** The link between the clinical/laboratory findings and MRE/CTE abnormalities needs to be corresponded when it comes to the diagnosis of intestinal vasculitis.

## Key points

- While gastrointestinal presentations are uncommon in vasculitis, their timely diagnosis is critical.
- Patients may present with a wide variety of non-specific findings on MRE/CTE.
- Extraintestinal findings are useful that should be covered in the MRE/CTE field.
- Imaging features need to be linked with the clinical and laboratory data.

## Background

Vasculitis is a term used for a heterogeneous group of conditions resulting in inflammation and injury of blood vessels in a variety of organs [1]. While this condition is rare, it can potentially have fatal outcomes due to resulting necrosis and ischemia. Therefore, there would be a need for early diagnosis and therapeutic interventions. Due to the non-specific clinical and imaging findings, diagnosis of vasculitis is often challenging. This is due to its similarities with other conditions, such as infections, malignancies, connective tissue diseases, and thromboembolic disorders [2–4]. A clinical history of systemic diseases, along with constitutional symptoms, including fever, weight loss, and fatigue, and most importantly, a dramatic response to immunosuppressive therapy could suggest a potential diagnosis of vasculitis.

A wide variety of vasculitides can present with abdominal manifestations, which could be the first presentation of a systemic disease [4–6]. While vasculitis of the gastrointestinal (GI) tract is rare, its diagnosis holds immense significance as late or missed recognition can result in high morbidity and mortality rates [5]. Abdominal pain, nausea, vomiting, diarrhea, GI bleeding (commonly presented as occult blood in stool, hematochezia, melena, and hematemesis), and weight loss are the most common GI presentations. Rarely, patients may develop serious complications, including intestinal perforation, ischemia, or peritonitis [7]. The wide spectrum of clinical and imaging findings in vasculitis, which may be non-specific and overlapping with other conditions, raises a diagnostic challenge.

Patients with vasculitis of the GI tract may undergo computed tomography/magnetic resonance enterography (CTE/MRE) to assess intestinal pathologies responsible for the non-specific clinical manifestations [7]. In addition to intestinal findings, CTE and MRE can often detect extra-intestinal pathologies [8–10]. The most common radiographic features in the GI tract, seen in different types and various stages of vasculitis, are submucosal edema or hemorrhage, mesenteric edema, bowel dilatation, and findings of acute or chronic mesenteric ischemia. Eventually, the ischemia may result in bowel perforation or stricture [4, 11].

Given the resemblance of radiologic findings with other entities such as infection, inflammatory bowel disease (IBD), and mesenteric ischemia secondary to vascular thromboembolism, diagnosis of vasculitis is often overlooked on MRE or CTE [12]. It is a diagnostic challenge and could not be simply differentiated from other disorders based on imaging findings alone. Therefore, a multidisciplinary team approach and histopathologic analysis are often required to make a definitive diagnosis. Radiologists must be aware of the diverse imaging patterns in intestinal vasculitis since they might be the first caregivers suggesting the proper diagnosis preventing misdiagnosis [12]. To the best of our knowledge, there is no comprehensive review describing the imaging characteristics of intestinal vasculitis on MRE/CTE.

Herein, we present radiologic findings of GI vasculitis with a focus on CTE and MRE by representative clinical cases. We highlight the diagnostic clues in clinical features and laboratory findings, helping in establishing the diagnosis. Lastly, a practical algorithm is provided to assist in the diagnosis of intestinal vasculitis and to differentiate it from other common GI disorders.

## Classification of vasculitis: an overview

**Fig. 1:**
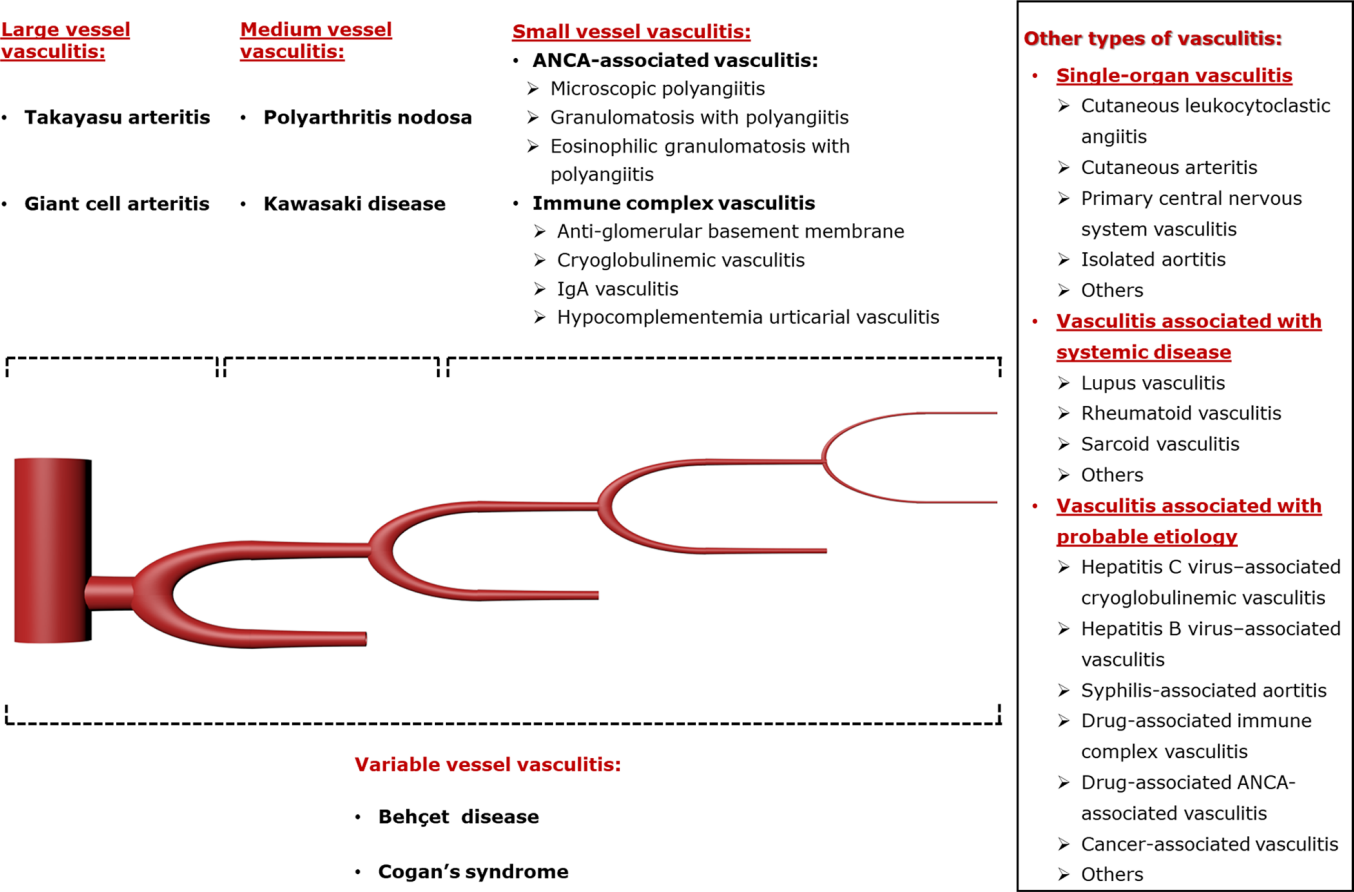
Diagram of various types of vasculitis based on the size of the affected vessel and etiology

Vasculitides are classified as (1) primary (idiopathic), with most likely autoimmune causes, and (2) secondary, which can stem from malignancy, infection, connective tissue diseases, drugs, or environmental exposures. The revised Chapel Hill Consensus Conference nomenclature system classifies vasculitides primarily according to the predominant type of vessel involvement and etiology. The classification is into seven categories: large-, medium-, small-, and variable-vessel vasculitis, single-organ vasculitis, vasculitis associated with systemic disease, and vasculitis associated with probable etiology (Fig. 1) [13]. Clinical presentation and imaging features are typically related to the size and location of involved vessels and the extent of the disease.

**Fig. 2:**
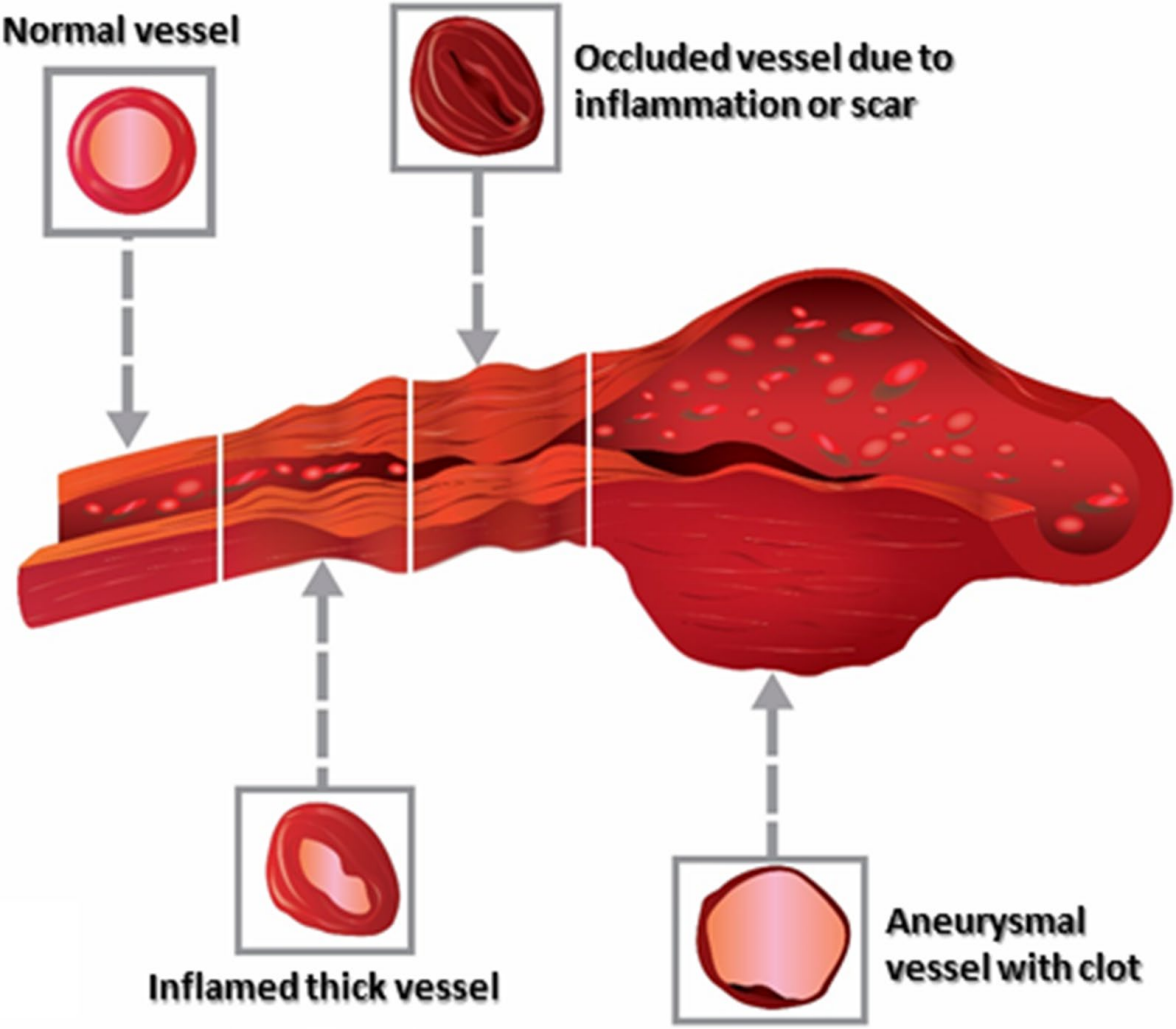
Schematic illustration on the spectrum of vasculitis related damage on the vessel wall

While the exact pathophysiology of vasculitis is not elucidated, the inflammation caused by infiltration of immune cells (lymphocytes, neutrophils, and monocytes/macrophages) in different layers of the vessel walls, release of pro-inflammatory cytokines, deposition of immune complexes, or presence of auto-antibodies can trigger vascular injury. Subsequent to the inflammation, conditions such as stenosis, occlusion, and rarely aneurysms can develop within the vessel (Fig. 2). Aneurysms commonly result from the destruction of the media layer and are associated with a risk of spontaneous hemorrhage and mural thrombosis. Obliteration or stenosis of the arterial lumen leads to decreased perfusion in abdominal organs, and in case vasa vasorum is involved, it can result in intestinal ischemia. Furthermore, diffuse intimal calcification may occur as a result of chronic vascular inflammation [14].

## Technical considerations

The 2017 consensus statement of joint European Society of Gastrointestinal and Abdominal Radiology (ESGAR) and European Society of Pediatric Radiology (ESPR) describes routine technical considerations for cross-sectional small bowel and colonic imaging [15]. Three main additional technical points should be incorporated into the routine MRE and CTE protocols when intestinal vasculitis is suspected:

(a) Arterial phase should be added to the routine post-contrast portal or enteric phase, which enhances visualization of arterial structures and vasculitis-associated intestinal ischemia [16, 17];

(b) As concurrent involvement of solid abdominal organs may be the only key to distinguish intestinal vasculitis from other entities, it is important to expand the field of scan as far as possible to cover extra-intestinal organs while avoiding a drop in image quality.

## Large-vessel vasculitis

Large-vessel vasculitides predominantly involve the great vessels, such as the aorta and its major branches. Takayasu arteritis and giant cell arteritis (GCA) are the most prevalent vasculitis in this subgroup. Considering the lack of specific diagnostic biomarkers, imaging modalities, including Doppler ultrasound, CT or MR angiography, digital subtraction angiography, and 18F-fluorodeoxyglucose positron emission tomography (PET), play a crucial role in diagnosing large-vessel vasculitis [1, 18, 19]. GI involvement is far more common in Takayasu arteritis than GCA. Radiologic features may be similar to mesenteric ischemia caused by thromboembolism or non-occlusive causes [20, 21]. The other differential diagnoses include atherosclerosis, connective tissue disorders, such as Marfan syndrome, Ehlers–Danlos syndrome, and Loeys–Dietz syndrome [12, 22].

### Takayasu arteritis

Takayasu arteritis is an autoimmune-mediated granulomatous inflammation of the aorta, its major branches, and pulmonary arteries, most commonly affecting young Asian women [23]. Lesions are typically close to the origin of the primary aortic branch [24]. At the disease onset or during acute phases, the presentations can be non-specific, including malaise, fever, weight loss, anorexia, myalgia, or arthralgias. Abdominal pain was observed in approximately 16% of patients [25]. However, more characteristic signs and symptoms develop with disease progression, including reduced or absent peripheral pulses, arterial bruits, limb claudication, blood pressure discrepancies between the arms due to stenotic or occlusive lesions, and hypertension [23].

GI involvement is rare in Takayasu arteritis. The intestine, spleen, and, rarely, the liver may undergo ischemic changes due to stenosis or occlusion of large- and medium-size GI arteries [6, 25]. Notably, in a cohort of 79 patients with Takayasu, only one patient developed mesenteric ischemia [25]. Approximately one-third of patients have involvement of the abdominal aorta or mesenteric circulation [26]. Kermani et al., in a cohort of 125 patients with Takayasu, found involvement of the abdominal aorta, mesenteric artery, and renal arteries in imaging of approximately 38%, 35%, and 20% of the patients, respectively [27]. The vascular involvements included arterial stenosis, thrombosis, and, rarely, aneurysms. Aortic dissection is very rare, and less than ten cases have been reported involving the abdominal aorta [28]. Notably, several studies have reported a higher prevalence of IBD among patients with Takayasu arteritis with evidence of genetic overlap [29, 30].

**Fig. 3:**
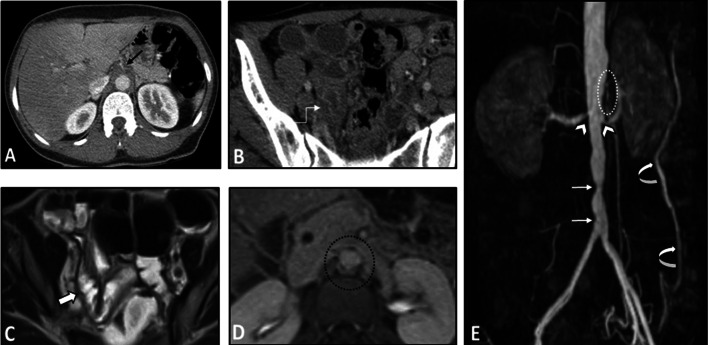
Intestinal vasculitis in a 35-year-old female with known Takayasu arteritis presenting with RLQ pain and elevated ESR. Axial contrast-enhanced CT scan (**A**) shows mural thickening and severe stenosis at the origin of the celiac artery (thin black arrow). Axial CT image (**B**) demonstrates circumferential mural thickening and submucosal edema at a single distal ileal segment (elbow arrow). MRE (**C**) was done following corticosteroid therapy after six days. Axial T2-W image shows complete resolution of corresponding ileal involvement (thick white arrow). Axial post-contrast T1-W image (**D**) displays concentric and irregular mural thickening and enhancement of abdominal aorta (black dotted oval) with double rings appearance (inner low and outer high enhancement). MR angiography (**E**) was performed. MIP image shows irregularity and multi-segmental stenosis at the distal abdominal aorta (thin white arrows). There is also bilateral stenosis at the origin of renal arteries (white arrowheads). The proximal segment of SMA shows irregularity and severe stenosis (white dotted oval). Abnormal dilatation of the arc of Riolan (white curved arrows) is evident, which supplies collateral flow to the SMA territory

The intestinal findings on the CTE/MRE may include segmental intestinal circumferential mural thickening, abnormal wall enhancement, and submucosal edema. The edema and enhancement reduce dramatically following corticosteroid treatment. Ischemic changes caused by vasculitis can be manifested by a diffuse long segment GI involvement [25]. Bowel dilation and mesenteric vascular engorgement are among other GI findings [12]. Extra-intestinal findings may include concentric mural thickening, transmural calcification, luminal stenosis, occlusion, aneurysmal changes, and collateral vessels formation in the abdominal aorta and its major branches, such as the celiac and superior mesenteric arteries. These vascular pathologies can also be seen on other cross-sectional imaging modalities. On contrast-enhanced CT, arterial mural enhancement can be found during the arterial phase, which is intensified during the late phase. However, since the measurement of mural enhancement may be hindered by the overshining intravascular contrast material, the black blood MRI technique is recommended for early detection of vessel wall inflammation [24]. Following contrast administration, the intima is poorly enhanced while the media-adventitia layer is intensely enhanced, resulting in a “double-ring” enhancement pattern (Fig. 3) [24, 31–33]. The possibility of underlying Takayasu arteritis should be considered when mural thickening or irregularity of the aorta and main branches is seen in younger age patients. Intestinal involvement in this disease may only cause a non-specific bowel wall thickening. Rapid response of bowel abnormality to steroid treatment which can be confirmed in follow-up imaging is a potential diagnostic clue.

### Giant cell arteritis

Giant cell arteritis (GCA) is a granulomatous vasculitis with a higher prevalence among women older than 50 and patients of northern European descent. GCA has a higher tendency to involve carotid, vertebral, and temporal arteries [34]. The disease manifestations include new-onset headache, jaw claudication, visual loss, constitutional symptoms, anorexia, and polymyalgia.

Abdominal involvement is extremely rare in GCA, especially when compared with Takayasu arteritis [35]. Kermani et al. reported mesenteric artery, abdominal aorta, and renal artery lesions in 17%, 6%, and 10% of patients, respectively [27]. So far, fewer than 15 cases of mesenteric ischemia have been reported [6], some with obstruction and infarction of the small intestine [36] or infarction of the sigmoid colon [37]. The abdominal aorta was involved in nearly half of GCA patients with aortitis, with approximately one-third presenting with abdominal pain [38]. Nevertheless, a recent longitudinal study found that abdominal aorta dilation in GCA was comparable to controls [39]. Acute abdominal pain in patients with GCA, which usually develops 6–7 years after disease onset, might also be an alarming sign for aneurysm or dissection [6, 40]. Strikingly, we did not identify any investigation on MRE or CTE findings of GCA in the literature.

## Medium-vessel vasculitis

Medium-sized vessels, mainly splanchnic arteries and their branches, are predominantly involved in medium-vessel vasculitis. The onset of vasculitis in medium vessels is more acute and necrotizing than in large-vessel [1]. This group includes polyarteritis nodosa and Kawasaki disease. GI involvement is more common in polyarteritis nodosa, with up to 95% of patients presenting with abdominal pain [1]. Radiologic findings include discovery of microaneurysms, arterial stenosis/occlusion, signs of intestinal ischemia, bleeding or perforation, rupture of hepatic, splenic, and/or renal (micro-)aneurysms, and rarely segmental hepatic or splenic infarction. Conventional angiography is the most reliable imaging technique utilized to investigate splanchnic vascular abnormalities [41, 42].

### Polyarteritis nodosa

Polyarteritis nodosa (PAN) is a systemic necrotizing vasculitis affecting medium and small vessels like in the kidneys without glomerulonephritis. It can occur in patients of any age, gender, or ethnicity; however, the peak incidence is at the 5th–6th decade of life. It involves the kidneys, skin, musculoskeletal system, nerves, and GI system [43]. The inflammation commonly affects all layers of vessels, resulting in stenosis, occlusion, or aneurysm. Therefore, tissue ischemia or hemorrhage may be observed in various organs, leading to acute life-threatening complications. Theoretically, histological confirmation of segmental fibrinoid necrosis of medium-sized vessels is required for the diagnosis. Nevertheless, the diagnosis can also be established based on clinical, laboratory (absence of antineutrophil cytoplasmic antibody (ANCA)), and radiologic findings. Angiography is the preferred imaging modality for detecting microaneurysm formations in visceral arteries [1, 43, 44].

PAN can cause heterogeneous GI manifestations, with non-specific abdominal pain occurring in 35–95% of the patients [4, 6, 11]. Other GI presentations are nausea, vomiting, diarrhea, gastroduodenal ulcers (mainly in the jejunum), hematochezia, hematemesis, and melena. Mesenteric vasculitis is observed in 50–60% of patients, which can lead to GI ischemia [26] or (rarely) aneurysm formation [45]. Serious abdominal complications include GI bleeding caused by ischemic mucosal ulcerations, bowel infarct or perforation, as well as perforation of microaneurysms, and organ ischemia due to visceral artery involvement [6, 46]. The severe manifestations rarely develop (< 5%) and can be a predictor of high mortality [11, 46]. Intestinal ischemia has even been reported as the initial presentation of the systemic disease [47]. PAN is the most common systemic vasculitis associated with gallbladder disease, with nearly 4% of patients developing cholecystitis [11]. While hepatic and splenic complications are rare, the patients may experience occlusion of hepatic veins (Budd–Chiari syndrome) and segmental liver or spleen ischemia [6, 7].

**Fig. 4:**
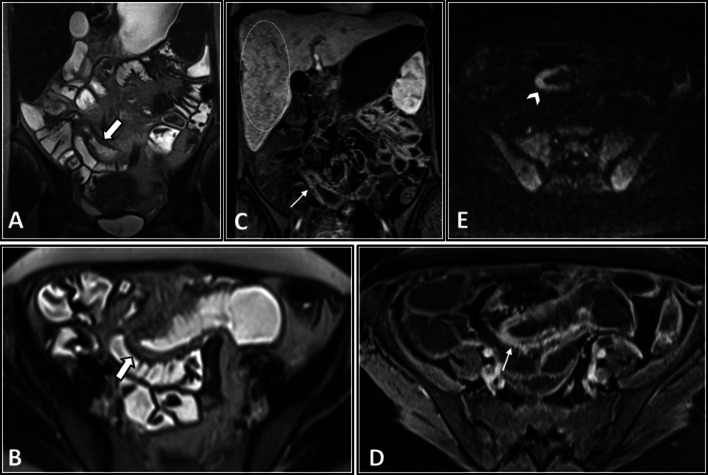
Intestinal vasculitis in a 32-year-old female with PAN (polyarteritis nodosa) presenting with episodic abdominal pain, fever, anemia, and elevated liver enzymes and inflammatory markers. MRE was obtained. Coronal and axial T2-W images (**A**, **B**) show a single thickened segment of mid ileum (thick white arrows) with mild T2 hyperintensity more likely secondary to submucosal edema. This is associated with mild degree of upstream bowel distention. Coronal and axial post-contrast T1-W images (**C**, **D**) display mural hyperenhancement (thin white arrows) with a relatively striated pattern. DWI at a b-value of 800 s/mm^2^ (**E**) shows restricted diffusion (white arrowhead), suggesting increased inflammatory cell density. The patient underwent double-balloon enteroscopy following this MRE, but it was complicated by bowel perforation at this segment. Then, surgical resection and ileostomy were performed. Histopathological assessment of resected segment revealed vasculitis consistent with polyarteritis nodosa (PAN). Heterogeneous enhancement of liver at arterial phase is also depicted (C), which raises suspicion for microvascular involvement (white dotted oval)

**Fig. 5:**
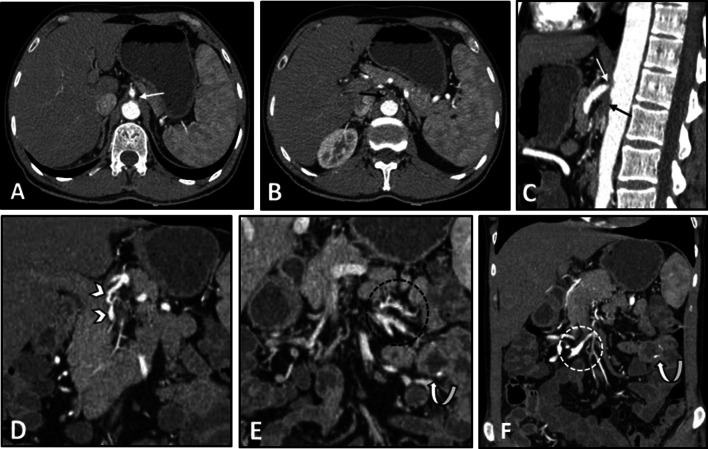
Vasculitis of splanchnic arteries in a 43-year-old male with a history of pathologically proven polyarteritis nodosa and segmental jejunal resection due to ischemia. He complains of postprandial abdominal pain, nausea, vomiting, hypertension, and elevated inflammatory markers. CTE was done. Axial and sagittal images (**A**–**C**) in the arterial phase demonstrate severe stenosis at the origin of the celiac trunk (thin white arrows) and complete occlusion and irregularity of proximal SMA (thin black arrows). Coronal CT images (**D**–**F**) reveal vessel irregularity and mild degree of aneurysmal dilations in a collateral branch between celiac trunk and SMA (white arrowheads), thickening and irregularity in jejunal branches of SMA (black dotted oval) adjacent to the previous site of jejunal resection and anastomosis (white curved arrows). The ileocolic branch of SMA also shows focal aneurysmal dilatation and mild thickening (white dotted oval)

The intestinal findings on CTE/MRE include segmental bowel mural thickening, submucosal edema, and abnormal hyperenhancement with striated pattern (Fig. 4). The involved intestinal region may also show diffusion restriction on DWI sequence. Luminal stenosis, mural irregularity and thickening, and aneurysmal dilation can be detected in visceral arteries, especially in the superior mesenteric artery (SMA) branches (Fig. 5). Visceral infarction and ruptured aneurysm may also be observed on contrast-enhanced CT [26, 48]. While microaneurysms (of renal, mesenteric, and/or splenic arteries) are the hallmark of PAN, they are not often directly detectable on enterography [49]. However, heterogeneous liver enhancement can indicate microvascular abnormalities (Fig. 4). Besides, the addition of an arterial phase in the imaging protocol may improve the detection of the microaneurysms. Since the kidney is the most commonly affected organ, its end-organ damage can present as striated nephrogram indicating renal infarct [49, 50]. Hydronephrosis resulting from detrusor muscle spasm is another urinary system complication [4, 51].

Differential diagnoses of splanchnic vascular abnormalities in PAN include segmental arterial mediolysis (SAM), fibromuscular dysplasia (FMD), and mycotic aneurysm. Intestinal involvement in PAN may mimic other inflammatory conditions like Crohn's disease (CD). A clinical history of underlying systemic disorders, a dramatic response to immunosuppressive therapy, and most importantly abnormalities of visceral arteries could suggest a potential diagnosis of vasculitis. In the absence of bowel abnormality, involvement of branch points in mesenteric vessels and sparing of the main renal artery are useful clues to distinguish vasculitis from SAM and FMD, respectively. In a young patient with clinical manifestation of mesenteric ischemia, a thorough inspection of visceral arteries should be done in the arterial phase of CTE/MRE to find segmental arterial wall thickening, stenosis, or aneurysmal dilatation, especially in the presence of constitutional symptoms, elevated levels of inflammatory markers showing a systemic inflammatory response (such as erythrocyte sedimentation rate (ESR) and C-reactive protein (CRP)). It is important to exclude vasculitis when mesenteric ischemia is not located in the classic watershed territory of bowel ischemia.

### Kawasaki disease

Kawasaki disease is a self-limiting acute necrotizing vasculitis affecting the medium and small vessels in almost all organs. Infants and children under five years of age and of Asian ethnicity are most commonly affected. Adults are scarcely affected, and they commonly have incomplete forms of the disease. GI manifestations include abdominal pain and/or distension, vomiting, diarrhea, paralytic ileus, jaundice, hepatomegaly, gallbladder hydrops, and, far less frequently, serious complications such as pancreatitis, GI obstruction, or pseudo-obstruction [6, 52–54]. In the largest series of patients with adult-onset Kawasaki disease, 56% had GI manifestations [55]. Presentation of GI involvement can be associated with a poor prognosis [52]. Notably, we did not identify any investigation on MRE or CTE features in Kawasaki disease.

## Small-vessel vasculitis

Small-vessel vasculitis predominantly involves small intraparenchymal vessels. This group based on pathogenesis is classified into two sub-groups; (1) antineutrophil cytoplasmic antibody (ANCA)-associated vasculitis (AAV) and (2) immune complex-associated vasculitis. Current imaging techniques due to limited resolution are unable to directly visualize small-vessel vasculitis. Consequently, imaging is used to assess the damaging effects on affected organs [56, 57]. Common findings on CTE/MRE include intestinal submucosal edema or hemorrhage, mesenteric edema, bowel dilatation, mesenteric fat haziness, and findings of acute or chronic mesenteric ischemia. Rarely, bowel perforation or stricture may occur as a consequence of inflammation. Differential diagnoses based on imaging include non-occlusive mesenteric ischemia, infectious enteritis, eosinophilic enteritis, IBD, radiation, and chemotherapy-induced enteritis.

### Antineutrophil cytoplasmic antibody (ANCA)-associated vasculitis

Antineutrophil cytoplasmic antibody (ANCA)-associated vasculitis (AAV) can occur at any age and includes granulomatosis with polyangiitis, microscopic polyangiitis, and eosinophilic granulomatosis with polyangiitis [58]. While AAV is a small-vessel vasculitis, it may cause inflammation of medium-sized vessels as well. A wide variety of organs, including the respiratory system, kidneys, nervous system, and GI tract, can be involved in AAV [59].

### Granulomatosis with polyangiitis (Wegener's granulomatosis)

Granulomatosis with polyangiitis (GPA) is a systemic necrotizing vasculitis of small- and medium-sized vessels with granulomatosis, which involves the upper and lower respiratory tracts and the kidneys. The condition is most common in the fourth or fifth decade of life [60]. Imaging can aid in determining the extent of disease, including pulmonary involvement [44]. The most common imaging findings in GPA are multiple and bilateral pulmonary nodules or masses [31, 61].

GI manifestations are present in 5–11% of patients with GPA. While any part of the GI tract might be involved, the abnormalities are commonly seen in the small and large bowel [6, 62]. GI manifestations include abdominal pain, ulceration (oral, esophageal, peptic, and intestinal), bloody diarrhea, peritonitis, and hematochezia. Rarely, patients may develop severe life-threatening intestinal involvement, including ischemia and perforation [6, 63]. Prolonged immunosuppressive treatment is postulated as the primary underlying risk factor for intestinal necrosis and perforation [63, 64]. The severe manifestations typically develop in patients with extensive involvement of the other organs shortly after the diagnosis and initiating the immunosuppressive therapy [63, 64]. Other rare abdominal complications of GPA may include granulomatous colitis, granulomatous pancreatic mass, splenic or hepatic infarct or hemorrhage, and gastritis [6, 65, 66].

**Fig. 6:**
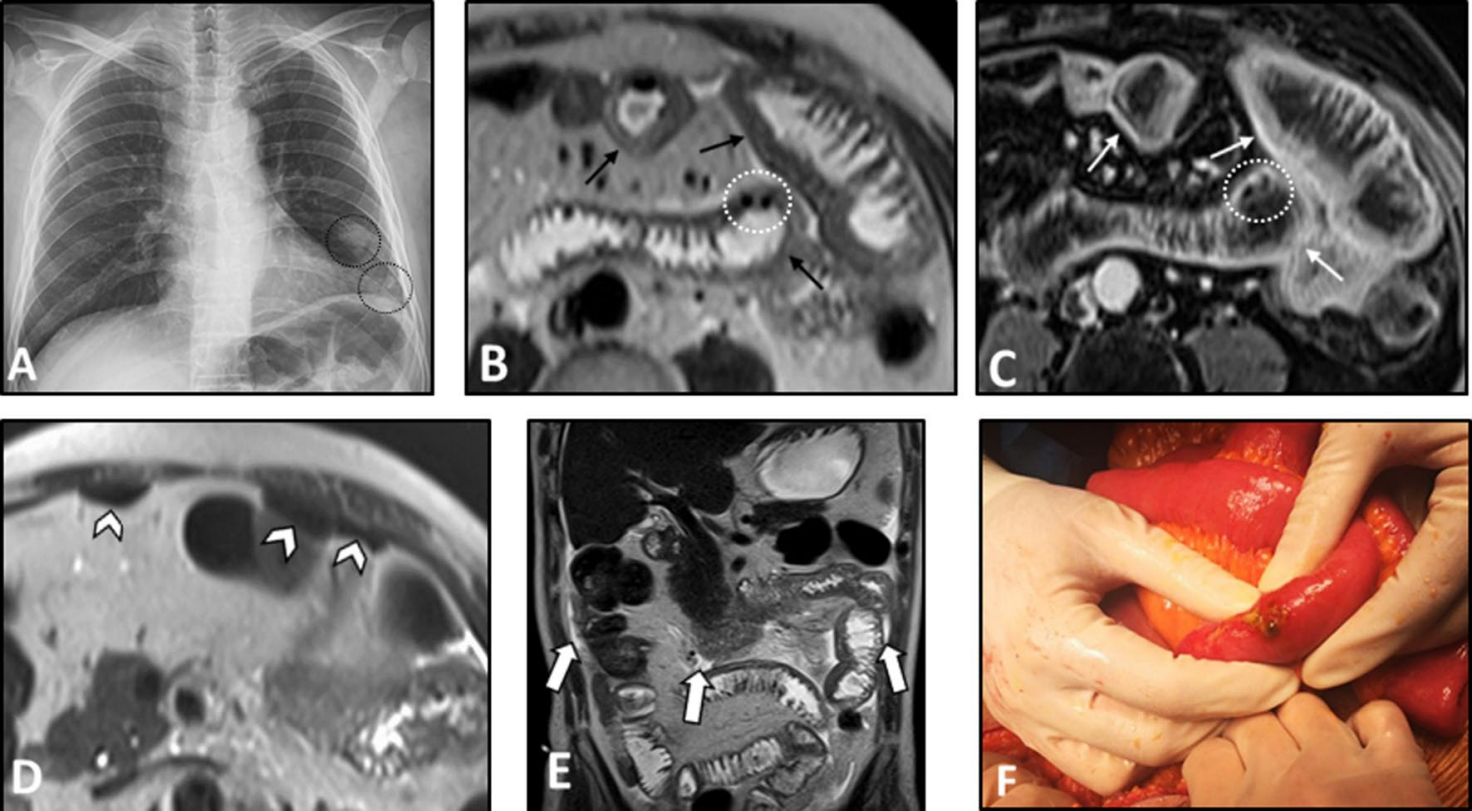
Intestinal vasculitis and perforation in a 56-year-old male with known ANCA-positive granulomatosis with polyangiitis and long history of immunosuppressive therapy presenting with acute abdominal pain and leukocytosis. Chest X-ray (**A**) shows two pulmonary nodules projecting over the lower zone of the left hemithorax (black dotted ovals). MRE was obtained. Axial T2-W image (**B**) reveals multi-focal segmental mural thickening in jejunal loops (thin black arrows). Axial post-contrast T1-W image (**C**) displays intense mural hyperenhancement (thin white arrows) in the involved segments. Focal intestinal pneumatosis (white dotted ovals) is also evident. Axial T2-W image (**D**) shows pneumoperitoneum (white arrowheads) at the non-dependent portion of the abdominal cavity, indicating bowel perforation. Coronal T2-W image (**E**) demonstrates small amount of free fluid (thick white arrows) at the abdominal cavity. Due to acute nature of presentation, the patient subsequently underwent laparotomy. Intraoperative photograph (**F**) reveals focal bowel necrosis and perforation. No signs of peritonitis were found in the initial examination, more likely due to the immunocompromised status of the patient

**Fig. 7:**
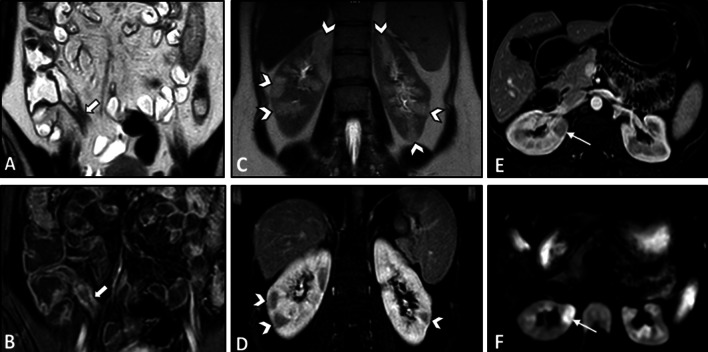
Intestinal vasculitis in a 39-year-old female presenting with episodic abdominal pain and findings of non-specific terminal ileitis in ileocolonoscopy examination. MRE was obtained. Coronal T2-W and post-contrast T1-W images (**A**, **B**) display mild mural thickening and hyperenhancement at distal terminal ileum (thick white arrows). Coronal T2-W and post-contrast T1-W images (**C**, **D**) reveal multiple peripheral wedge-shaped hyperintense areas at both kidneys, showing decreased enhancement on delayed phase (white arrowheads). Axial post-contrast T1-W and corresponding DWI images (**E**, **F**) show a hypoenhancing lesion at the right renal cortex with diffusion restriction (thin white arrows). Vasculitis was suspected as the underlying cause. Afterward, positive results for antineutrophil cytoplasmic antibody (ANCA) and inflammatory markers were consistent with the diagnosis of ANCA-associated vasculitis

**Fig. 8:**
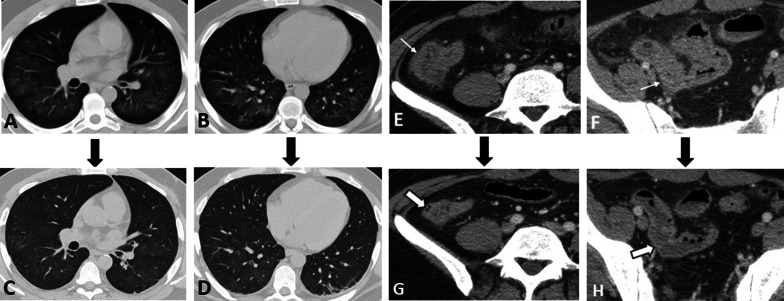
Intestinal vasculitis and pulmonary alveolar hemorrhage in a 41-year-old male with ANCA-positive granulomatosis with polyangiitis (GPA) presenting with abdominal pain and hemoptysis. Axial thoracic CT images (**A**, **B**) at two different levels show diffuse mosaic attenuation secondary to geographic areas of ground-glass opacity, suggestive for alveolar hemorrhage. Follow-up CT images (**C**, **D**) three days after corticosteroid therapy demonstrate a notable response to treatment. CTE was also done with suboptimal small bowel distention (**E**, **F**) at admission to assess the cause of abdominal pain that revealed circumferential mural thickening at ascending colon and distal ileum (thin white arrows) associated with adjacent mesenteric fat haziness. Follow-up CT images (**G**, **H**) show complete response to corticosteroid therapy with normal bowel wall thickness (thick white arrows) and a decrease in attenuation of the ileocolic mesenteric root

Multi-focal segmental circumferential mural thickening and abnormal hyperenhancement of the small and/or the large intestine can be detected on MRE/CTE (Fig. 6). Mesenteric vascular engorgement or unexpected large-vessel pathology (i.e., stenosis or dilation) and ascites can be observed as well [4, 51, 67]. Mesenteric fat haziness is another occasional finding, which stems from inflammation of the mesenteric vessels. In patients presenting with acute abdominal pain, signs of bowel perforation and/or necrosis, including focal intestinal pneumatosis and mild ascites, can be present. However, it should be noted that the immunosuppressive treatment might mask acute symptoms of intestinal ischemia (Fig. 6). The presence of pathologies in the kidneys can be a valuable diagnostic clue. Wedge-shaped T2-weighted hyperintensities and hypoenhancing lesions at the renal parenchyma with diffusion restriction on DWI are potential findings that can imply interstitial nephritis or renal ischemia (Fig. 7). Splenic involvement, including splenomegaly and splenic infarction, can also be detected [51]. In patients with bowel pathologies, concurrent abnormal laboratory results or imaging findings related to pulmonary or renal involvement and positive c-ANCA titer are helpful clues to differentiate GPA from other disorders (Fig. 8).

### Microscopic polyangiitis

Microscopic polyangiitis (MPA) (also known as leukocytoclastic vasculitis) is a systemic autoimmune non-granulomatous vasculitis of the small vessels associated with the presence of ANCA, with a slightly higher prevalence in men [68]. Similar to PGA, kidneys and lungs are the primary organs that are involved. However, skin lesions (commonly present as palpable purpura), neurological manifestations, and GI involvement may also occur.

GI involvement is observed in 5–30% of the patients [6, 68]. Abdominal pain is the most frequent GI presentation, associated with nausea, vomiting, and diarrhea. Lower GI bleeding and ischemia are rare but life-threatening complications [46, 68–70], which can scarcely result in massive hemorrhage [71].

**Fig. 9:**
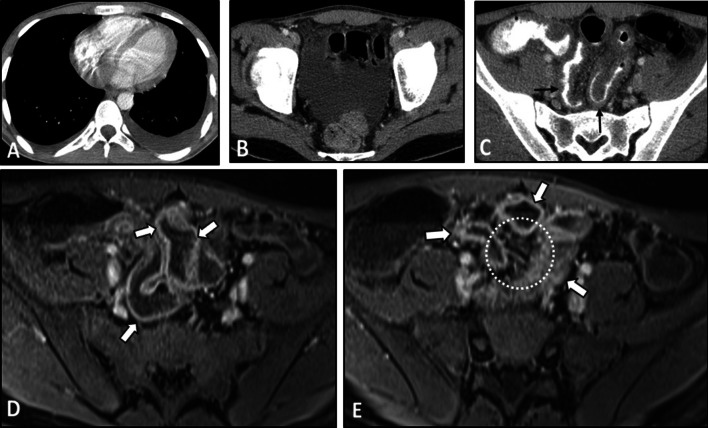
Intestinal vasculitis in a 28-year-old male with leukocytoclastic vasculitis presenting with abdominal pain, chest discomfort, and erythematous rashes with palpable purpura on the lower extremities. An abdominopelvic CT scan with IV and oral contrast was obtained. Axial images (**A**, **B**) show mild bilateral pleural effusion and pelvic free fluid. Also, axial CT (**C**) demonstrates mild concentric mural thickening of the distal ileal loop (thin black arrows). The patient underwent a biopsy of skin lesions. The histopathologic study revealed leukocytoclastic vasculitis. Corticosteroid therapy started. Then, MRE was done after five days. Axial post-contrast T1-W images (**D**, **E**) display mild mural thickening and hyperenhancement (thick white arrows) in the distal ileal segments. Mild engorgement of adjacent mesenteric vessels (white dotted oval) is also evident, and there is no ascites. Imaging findings and clinical data were indicative of an excellent response to therapy

Radiologic features of bowel involvement are similar to other small-vessel vasculitides [4]. Correspondingly, intestinal concentric mural thickening and post-contrast hyperenhancement can be detected on enterography (Fig. 9). Engorgement of adjacent mesenteric vessels is one of the other potential findings. In acute cases, signs of bowel infarction or perforation can be rarely observed [4]. Similar to GPA, the presence of extra-intestinal renal and pulmonary pathologies can be an important diagnostic clue. Cutaneous lesions, including palpable purpura, especially on the extremities, may be the key clinical feature of leukocytoclastic vasculitis compared to similar conditions.

### Eosinophilic granulomatosis with polyangiitis (Churg–Strauss)

Eosinophilic granulomatosis with polyangiitis (EPGA) is a necrotizing granulomatous small-vessel vasculitis characterized by adult-onset asthma and tissue eosinophilia. Patients can be divided into two groups: (1) ANCA-positive patients (30–40%) with predominant vasculitis manifestations, and (2) ANCA-negative patients with predominant eosinophilic presentations [72]. The most common signs and symptoms include asthma, lung infiltrates, neuropathy, and constitutional symptoms, while cardiac, skin, renal, and GI involvement are less frequent [72].

GI presentations can be observed in 30–50% of patients, including abdominal pain, diarrhea, melena, and hematochezia. No significant difference is found in the frequency of GI manifestations between ANCA-positive and ANCA-negative patients [72]. Vasculitis of the mesenteric artery is the most prevalent cause of GI involvement, resulting in bowel ischemia. Mucosal eosinophilic infiltration can be another underlying cause of ulceration, motility disorders, and obstructive symptoms [6]. Mucosal ulcers, which are typically limited to the small intestine, can be detected by endoscopy [72]. Severe complications, including bowel obstruction and stenosis, can occur in 22–45% of the patients [72]. Hepatobiliary complications are infrequent and include cholestasis and liver infarction [73–75]. The potential underlying causes of hepatic involvement are fibrinoid necrosis of arteries, eosinophilic infiltration, and granulomatous reaction in the portal area. These conditions may be seen in addition to the irregular narrowing of the small hepatic arteries, which can be detected by angiography [74].

**Fig. 10:**
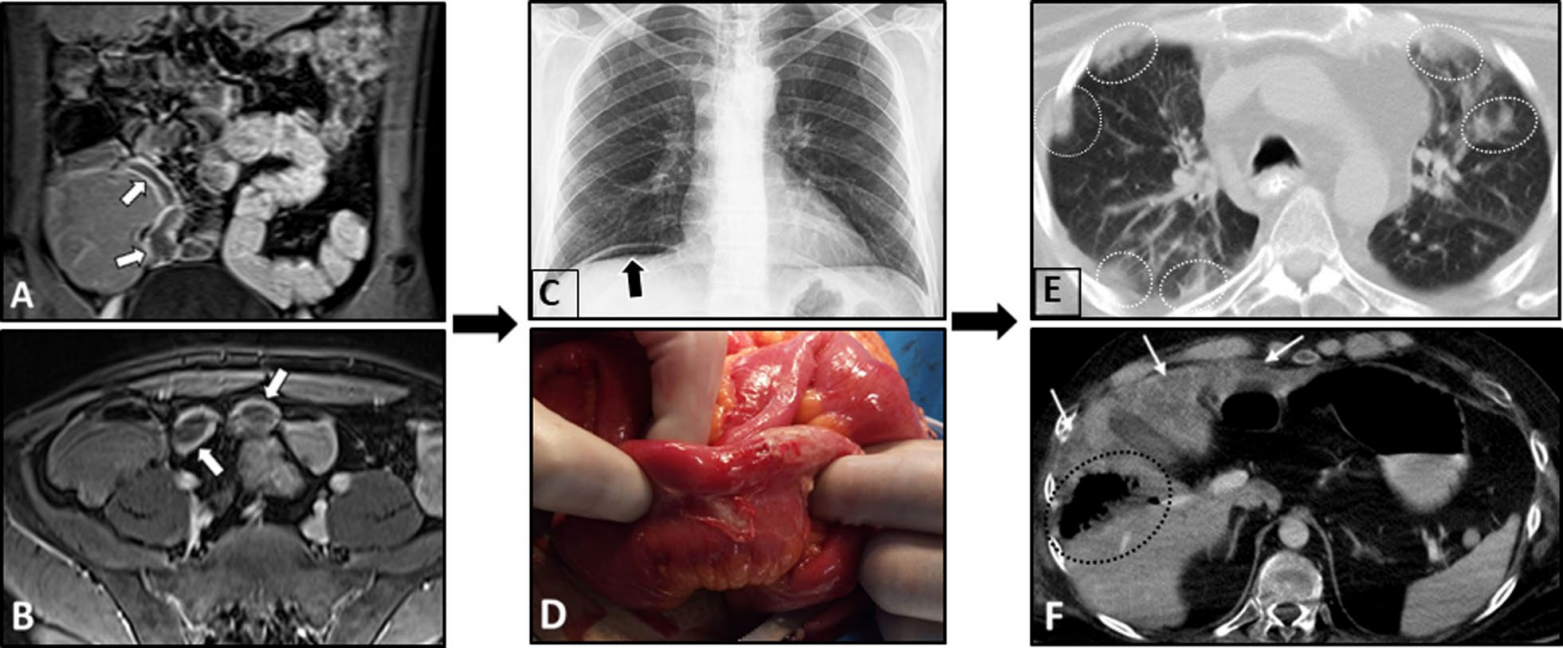
Intestinal vasculitis and perforation in a 32-year-old male with a history of asthma presenting with abdominal pain and eosinophilia. MRE was obtained. Coronal and axial post-contrast T1-W images (**A**, **B**) display increased mural enhancement at the terminal ileum without thickening (thick white arrows). Three months later (**C**, **D**), the patient presents to the ER with acute abdominal pain without definite diagnosis or treatment. Chest X-ray (**C**) shows subdiaphragmatic free gas (thick black arrow). He underwent an emergency laparotomy. Intraoperative photograph (**D**) revealed focal bowel perforation at the distal ileal segment. After segmental bowel resection, an ileostomy was performed. Eight months after surgery (E, F), he was admitted for RUQ pain and dyspnea. Axial chest CT image (**E**) demonstrates bilateral GGO and consolidations with lobular distribution (white dotted oval). Axial contrast-enhanced CT image (**F**) shows multiple wedge-shaped areas of hypoattenuation in the liver (thin white arrows), consistent with hepatic infarction. Intraparenchymal gas formation is also evident at the infarcted segment, suggestive for abscess formation (black dotted oval). Small-vessel vasculitis was confirmed based on clinical/laboratory data and pathology results suggestive of Churg–Strauss syndrome. Unfortunately, he died secondary to sepsis

On MRE, intestinal mural hyperenhancement can be delineated, which may or may not be associated with mural thickening (Fig. 10) [76]. Mural hyperenhancement can also be evident on CT images [72], which occurs as a result of mesenteric vasculitis or mucosal eosinophilic infiltrations [4]. Submucosal edema causes a striated bowel wall appearance with diffuse mucosal enhancement manifesting as a halo sign [51]. Signs of severe complications include bowel obstruction and stenosis [77–79], and as a result, bowel dilatation is a frequent CT finding [72]. Hepatic infarction may present as multiple wedge-shaped areas of hypoattenuation. Moreover, signs of eosinophilic abscess, such as intraparenchymal gas and fluid formation, may also be evident [80]. Approximately 70% of patients with EGPA present with pulmonary involvement, such as patchy, transient, and non-segmental opacities that have no predilection for any lung zone. Since intestinal vasculitis related to EGPA could only present as increased wall enhancement on MRE/CTE without gross mural thickening, attention should be paid beyond the GI tract searching for important clues such as infarction of the visceral organs or lung involvement, particularly in patients with a history of asthma and eosinophilia [81].

### Immune complex-associated vasculitis

Similar to AAV, medium-sized vessels can be involved in addition to small vessels in immune complex-associated vasculitis. Activation of complements by the immune complexes leads to the attraction of neutrophils to the vessel wall and subsequently inflammation and vascular lesion. Radiologic findings are similar to other small-vessel vasculitides. This group includes immunoglobulin (Ig) A vasculitis and cryoglobulinemic vasculitis.

### IgA vasculitis (Henoch–Schönlein purpura)

IgA vasculitis, formerly called Henoch–Schönlein purpura (HSP), is a systemic, leukocytoclastic vasculitis affecting small vessels. Being the most common vasculitis in children, IgA vasculitis in adulthood is less prevalent than in childhood. While the etiology is not well understood, genetic and environmental factors play a major role in disease pathogenesis. The most common manifestations are palpable purpura, arthralgia and/or arthritis, glomerulonephritis, and acute enteritis [82].

GI involvement can be observed in approximately two-thirds of patients. Abdominal pain is the most common GI presentation, followed by nausea, vomiting, melena, and/or rectorrhagia [83]. In 40–50% of the patients, the mesenteric circulation is involved [26]. Rarely (3–5%) patients may experience serious complications, including bowel infarct, perforation [84], obstruction, or irreducible intussusception [85]. Intestinal ischemia can even be the initial manifestation of the disease (29). Seldom (< 5%) the hepatobiliary system can be involved as well [86].

**Fig. 11:**
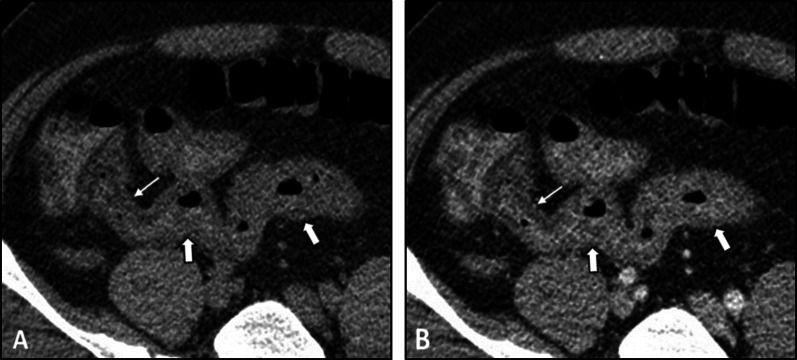
Intestinal ischemia in a 37-year-old male with a history of known Henoch–Schönlein purpura (HSP) presenting with abdominal pain and bloody diarrhea. The patient underwent pre (**A**)- and post (**B**)-IV contrast CT scans using positive dilute oral contrast. Segmental mural thickening (thick white arrows) is seen in the terminal ileum with a focal area of mural hypoenhancement (thin white arrows) proximal to the ileocecal valve. Ileocolonoscopy confirmed mucosal ischemic changes, and signs/symptoms were improved following treatment by corticosteroid

On imaging, signs of bowel ischemia and edema can be observed, including segmental mural thickening and post-contrast focal mural hypoenhancement (Fig. 11). However, hemorrhage is commonly limited to mucosa and submucosa and is self-limiting [4, 87]. Importantly, glucocorticoids, which are used to alleviate clinical symptoms such as arthralgia, can mask pain and signs of bowel ischemia [88]. Descending duodenum and the terminal ileum are frequently involved [89]. Dramatic improvement after corticosteroid therapy is a characteristic feature of GI involvement [89]. Gallbladder wall thickening can also be observed in 25% of those with hepatobiliary involvement [86]. The diagnosis of HSP is mainly based on typical clinical signs and symptoms like skin rash, arthritis, colicky abdominal pain, GI bleeding, and hematuria. Imaging characteristics of the bowel wall are also helpful in diagnosis and management. Based on our clinical experience, while mural hyperdensity in non-contrast CTE images favors submucosal hemorrhage, focal hypoenhancement in post-contrast images suggests ischemic changes or edema associated with inflammation.

### Cryoglobulinemic vasculitis

Cryoglobulins are abnormal immune system proteins that can deposit in the small vessel walls leading to cryoglobulinemic vasculitis. The skin, kidneys, joints, and peripheral nervous system are commonly involved. Type I cryoglobulinemic vasculitis is usually related to an underlying lymphoproliferative disorder. Types II (monoclonal) and III (polyclonal) are typically associated with rheumatoid factor activity. The main underlying causes of cryoglobulins production include lymphoproliferative disorders, systemic lupus erythematosus (SLE), Sjögren syndrome, and rheumatoid arthritis [90].

GI involvement is rare but can become disastrous. These manifestations include abdominal pain, melena, and more severe complications, such as intestinal perforation, intestinal ischemia, acute cholecystitis, and pancreatitis [91]. Liver involvement can be observed in up to 60% of the patients [6].

## Variable-vessel vasculitis

The vasculitis does not have any predominant vessel size or type involvement. This group includes Behçet's disease and Cogan syndrome [13]. In the latter syndrome, only a few cases of mesenteric vasculitis have been reported [92]. The location, extent, and size of the involved vessels determine the radiologic and clinical signs. Differential diagnoses of MRE/CTE findings are non-occlusive mesenteric ischemia, infectious enteritis, eosinophilic enteritis, IBD, radiation enteritis, and chemotherapy-induced enteritis.

### Behçet disease

Behçet's disease is a systemic inflammatory vasculitis affecting vessels of all sizes and also multiple organs. Ocular lesions, oral aphthous ulcers, genital ulcers, and skin lesions are the characteristic manifestations, which can be associated with vascular, neurological, and GI involvement. The prevalence of Behçet's disease is higher in the Mediterranean, Middle East, and the Far East regions (along the ancient Silk Route) and is associated with the distribution of HLA-B51. The disease onset is commonly at the third or fourth decades of life [93].

The prevalence of GI manifestations is widely varied in different geographic regions, ranging from 1.4 to 60% [94–96]. GI involvement is more frequent in the Far East than in the Middle East and Europe. Abdominal pain, usually in the right lower quadrant, is the most common presentation, followed by diarrhea and GI bleeding [96]. In Behçet's disease, GI complications typically have a relapsing nature [97]. The ileocolic region, including the ileocecal area, ileum, or different colonic segments, is most commonly involved [98, 99]. Bleeding and perforation are among serious complications; however, in chronic cases, closed perforation might develop. Acute lower GI bleeding has been reported in approximately 11–25% of those with GI manifestations, and it might be the initial GI presentation [100, 101]. In addition to intestinal involvement, thrombosis of the hepatic vein or inferior vena cava can result in hepatic complications, particularly Budd–Chiari syndrome. Very rarely, large-vessel involvement might result in the formation of abdominal aorta aneurysms [102].

**Fig. 12:**
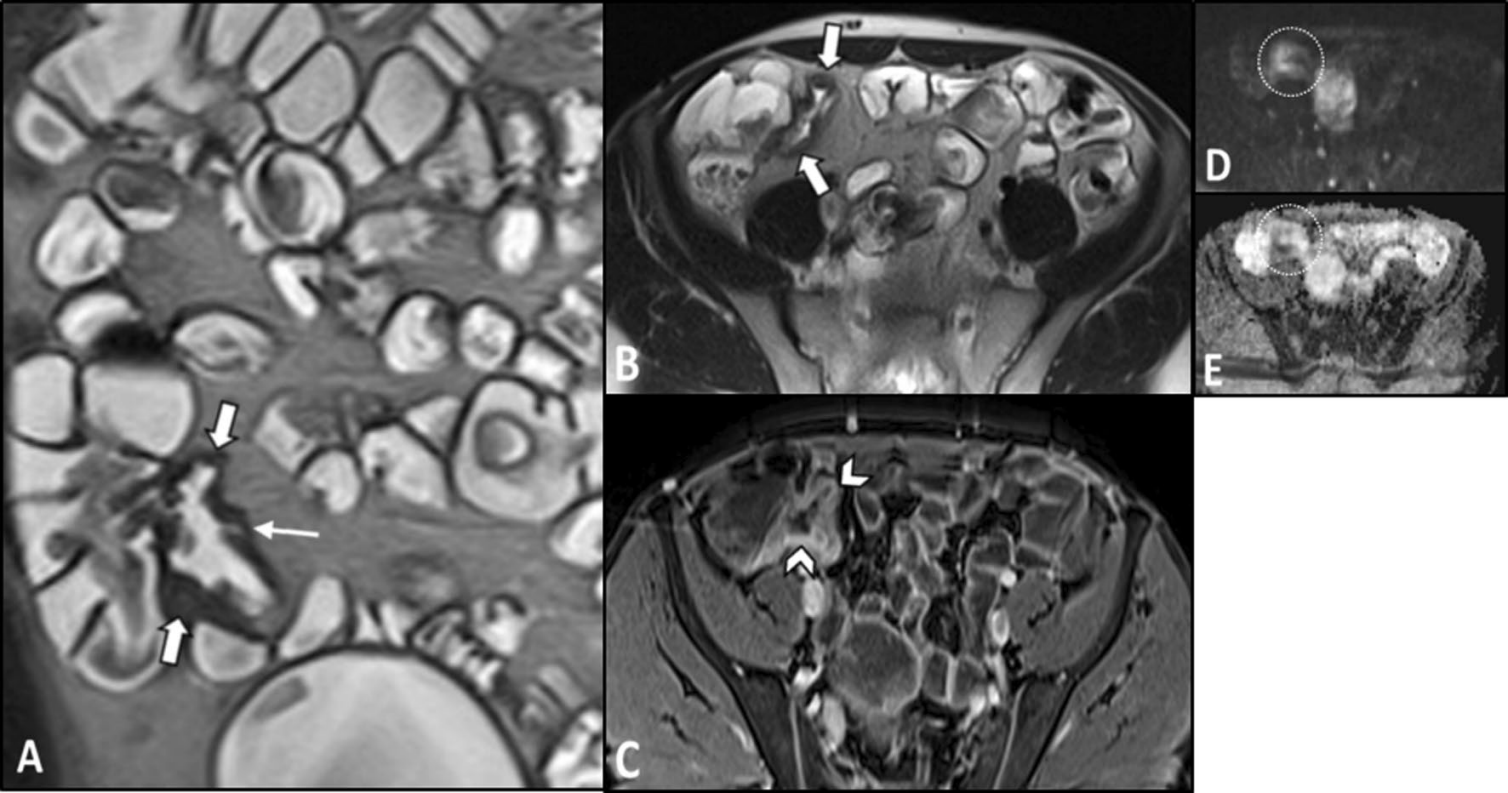
Terminal ileitis in a 23-year-old female with a history of recurrent oral and genital aphthous ulcers and uveitis presenting with RLQ pain finally diagnosed as Behçet’s disease. MRE was obtained. Coronal and axial T2-W images (**A**, **B**) show mural thickening in the terminal ileum (thick white arrows) without significant predilection to the mesenteric side in contrast to Crohn’s disease. There is also a deep penetrating ulcer (thin white arrow). Axial post-contrast T1-W image (**C**) displays increased contrast enhancement in the thickened terminal ileal segment (white arrowheads) without surrounding mesenteric inflammatory changes. Axial DWI image and corresponding ADC map obtained at b-value of 800 s/mm^2^ (**D**, **E**) show restricted diffusion in the involved segment (white dotted oval). Histopathologic examination confirmed Behçet’s disease

**Fig. 13:**
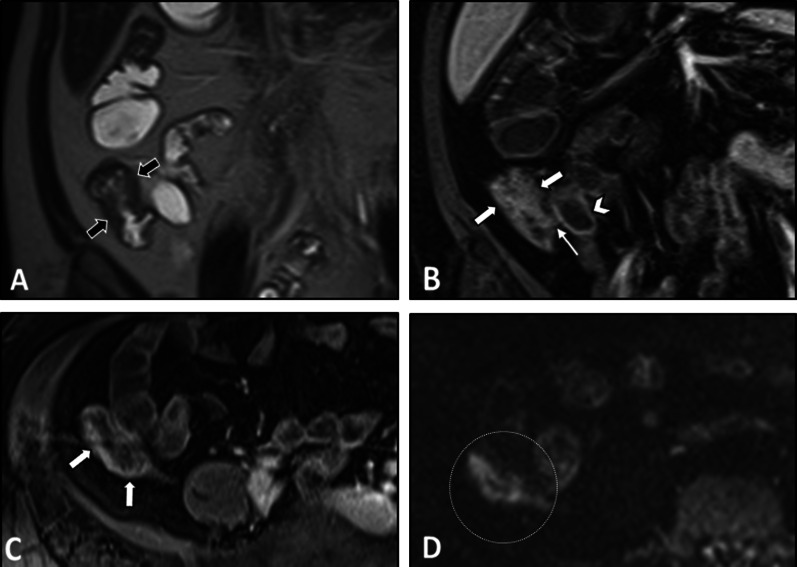
Intestinal vasculitis in a 26-year-old female with known Behçet’s disease and positive HLA-B5 complaining of episodic RLQ pain. MRE was obtained. Coronal T2-W image (**A**) demonstrates irregular circumferential mural thickening of the cecum and proximal ascending colon (thick black arrows) without mesenteric side predilection or pericolic infiltration. Coronal and axial post-contrast T1-W images (**B**, **C**) show marked enhancement of thickened colonic wall (thick white arrows) mildly extending to ileocecal valve (thin white arrow) with low-grade mural hyperenhancement in the adjacent terminal ileum (white arrowhead). Axial DWI image (**D**) obtained at a b-value of 800 s/mm^2^ reveals restricted diffusion in the thickened cecum (white dotted oval)

On imaging, irregular circumferential mural thickening with homogeneous mural enhancement is the most common finding of bowel involvement. Behçet's disease can significantly mimic CD on MRE. Deep penetrating ulcers and restricted diffusion on DWI in the involved segment could also be observed (Fig. 12) [20, 103]. Unlike CD, there is no specific predilection to the mesenteric side in Behçet's disease with less surrounding mesenteric inflammatory changes (Fig. 13). Peker and colleagues reported a specificity of 100% and sensitivity of 57% for polypoid patterns and homogeneous mural enhancement findings in distinguishing Behçet's from CD [104]. While pathologies of more proximal ileal segments favor small bowel CD, ileocecal involvement favors Behçet's disease. Additionally, patients with Behçet's disease may have a shorter length of the involved segment than CD [104]. History of recurrent oral and genital ulcers and uveitis is also important to make the diagnosis. Mural enhancing saccular pseudoaneurysms of the abdominal aorta may also be detected [40].

## Vasculitis associated with systemic diseases

According to the revised Chapel Hill Consensus Conference nomenclature system, vasculitides secondary to connective tissue and autoimmune diseases are classified as vasculitis associated with systemic diseases [13]. While small vessels are commonly involved, medium and large vessels may also be affected. SLE, rheumatoid arthritis, systemic sclerosis (scleroderma), and antiphospholipid antibody syndrome are among diseases associated with vasculitis that can have concurrent GI involvement [6, 105–107].

### Systemic lupus erythematosus (SLE)

**Fig. 14:**
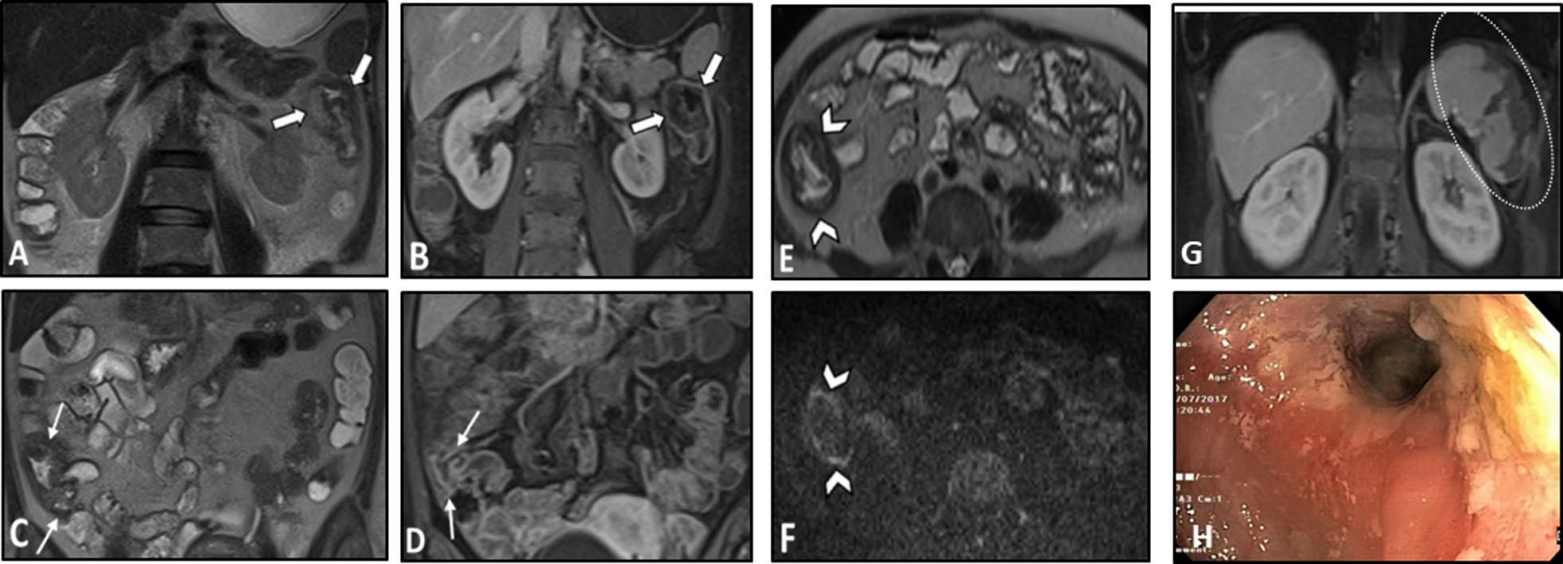
Intestinal ischemia in a 31-year-old female with known SLE-Sjögren overlap syndrome presenting with fever, anemia, and LUQ pain. MRE was obtained. Coronal T2-W and post-contrast T1-W images (**A**, **B**) display mural thickening, mucosal hypoenhancement, and serosal hyperenhancement of descending colon adjacent to the splenic flexure (thick white arrows) suggestive for mucosal ischemic changes. Coronal T2-W and post-contrast T1-W images (**C**, **D**) display mural thickening and increased mural enhancement of the cecum and ascending colon (Thin white arrows). Axial T2-W image and corresponding DWI sequence (**E**, **F**) show concentric mural thickening of the proximal ascending colon, demonstrating restricted diffusion (white arrowheads), in favor of ischemic changes or increased inflammatory cells. Coronal post-contrast T1-W image (**G**) reveals subcapsular areas of splenic hypoenhancement (white dotted oval), indicative of infarction. Pale and fragile mucosa is seen in colonoscopic view (**H**) of the involved colonic splenic flexure

SLE is a complex systemic disease with a female predominance resulting from immune complex deposition and production of auto-antibodies in various organs. The prevalence of vasculitis in patients with SLE ranges from 11 to 36%. Lupus vasculitis has various clinical manifestations as a result of involving vessels of all sizes [108, 109]. However, small-sized vessels are more frequently affected, and mesenteric vasculitis, also known as lupus enteritis, is far less common, with a prevalence of 0.2% to 9.7%, mostly involving the SMA [110]. SMA involvement can result in ileal and jejunal ischemia [108]. Lupus mesenteric vasculitis commonly presents with acute abdominal pain with diffuse localization [110]. Additionally, thrombosis of the mesenteric arteries may also occur even in the absence of vasculitis [111]. Rarely, GI manifestations, such as intestinal ischemia, can be the initial presentation of the systemic disease [47]. Notably, secondary Sjögren's syndrome may occur in approximately 14%–17.8% of SLE patients (Fig. 14) [112].

Imaging is an indispensable tool in the assessment of lupus enteritis. Multi-focal segmental circumferential wall thickening, submucosal edema, and mucosal ischemic changes are among the potential intestinal findings on MRE/CTE. Bowel wall thickening commonly caused by edema can result in a “thumb printing sign” presentation that can be found in bowel ischemia [113, 114]. The diffuse circumferential wall thickening with submucosal edema gives rise to a “target sign” or “double-halo” finding, which can help in distinguishing SLE from other pathologies [115, 116]. The local hypervascular appearance of the adjacent mesentery, often seen in CD, also known as the “Comb sign,” is one of the other findings on CT of patients with lupus enteritis. Collectively, comb sign and/or target signs are present in approximately 70% of the patients with lupus enteritis [117].

**Fig. 15:**
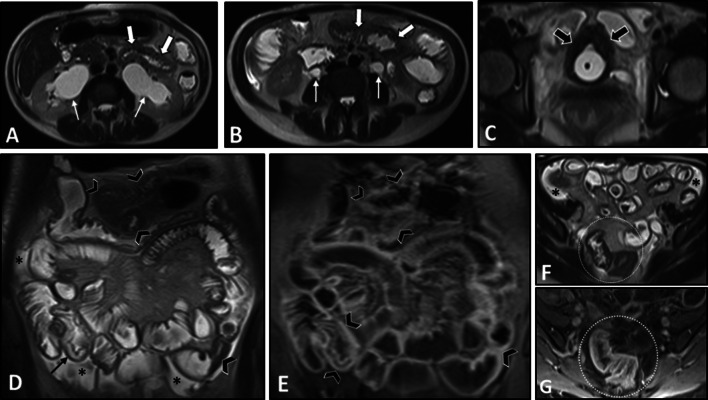
Intestinal vasculitis in a 42-year-old female with a known history of SLE and hypothyroidism presenting with generalized abdominal pain, fever, anemia, and elevated inflammatory markers. She also complained of dysuria, hematuria, and frequency. MRE was obtained. Axial T2-W images (**A**, **B**) show bilateral hydroureteronephrosis (thin white arrows) and multi-focal segmental circumferential mural thickening involving several jejunal and ileal loops (thick white arrows). Axial T2-W image (**C**) displays an under distended urinary bladder containing a Foley catheter showing diffuse wall thickening (thick black arrows). Coronal T2-W and post-contrast T1-W images (**D**, **E**) demonstrate multi-segmental mural thickening involving several jejunal and ileal loops showing increased enhancement due to active nature of inflammation (black arrowheads). Submucosal edema is also noted (thin black arrow). Axial T2-W and post-contrast T1-W images (**F**, **G**) also reveal involvement of rectosigmoid colon (white dotted ovals). Small amount of free fluid is noted at the abdominopelvic cavity (black asterisks)

**Fig. 16:**
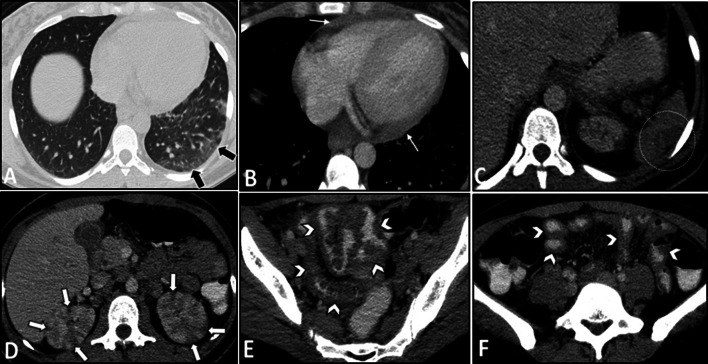
Intestinal vasculitis and renal infarction in a 29-year-old female with known SLE presenting with fever, malaise, generalized abdominal pain, dyspnea, and elevated ESR. An abdominopelvic CT scan with IV and oral contrast was done. There are subpleural patchy areas of GGO at the left lower lobe suggestive of lupus pneumonitis (thick black arrows in **A**). Small pericardial effusion and thickening (thin white arrows in **B**) are seen in favor of serositis. There is a wedge-shaped subcapsular hypodensity in the spleen (white dotted oval in **C**), indicative of splenic infarction. Multiple bilateral wedge-shaped renal parenchymal infarcts are also depicted (thick white arrows in **D**). Diffuse circumferential wall thickening of ileal loops (white arrowheads in **E** and **F**) is evident, representing lupus enteritis

**Fig. 17:**
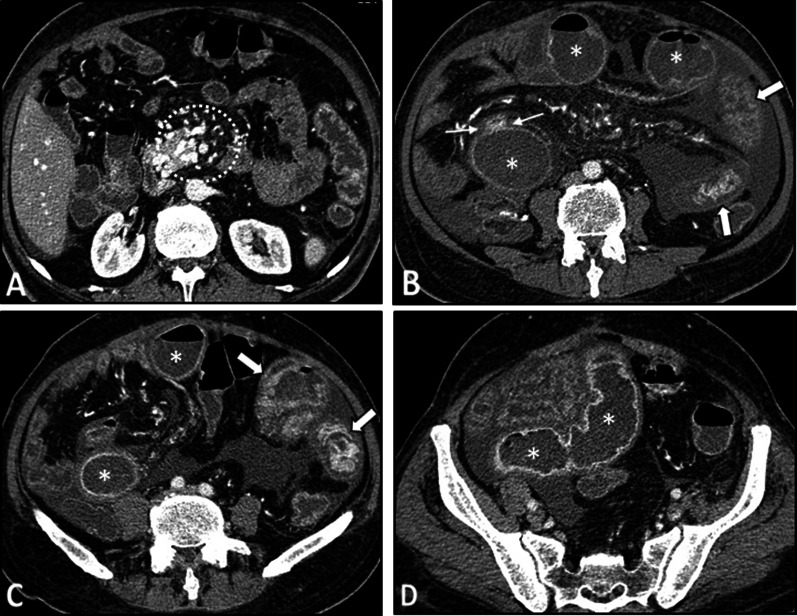
Bowel stricture with secondary bacterial overgrowth in a 44-year-old male with a history of primary antiphospholipid antibody syndrome. He presented with abdominal pain, fever, nausea, vomiting, and diarrhea without a history of previous surgery. CTE was obtained. Axial CT (**A**) shows evidence of chronic SMV thrombosis associated with multiple collateral veins in the mesenteric root (white dotted oval). CT images (**B**–**D**) demonstrate diffuse mural thickening at jejunal loops with submucosal edema and mucosal hyperenhancement (thick white arrows). There is an abrupt stricture in a segment of proximal ileum (thin white arrows) leading to upstream bowel dilatation (white asterisks) suggestive of partial obstruction with development of ascites. The clinical and imaging findings were suspicious for focal stricture secondary to an old vascular insult with secondary bacterial overgrowth in dilated jejunal and proximal ileal loops. The patient underwent antibiotic therapy, bowel rest, and conservative management resulting in improvement of signs/symptoms. He was discharged with an uneventful follow-up

The genitourinary system is frequently involved in SLE presenting as hydronephrosis, cystitis, and lupus nephritis, which could be used to reach the diagnosis (Fig. 15). Vasculitis should always be considered in patients with enteritis accompanied by single- or multi-organ infarcts (Fig. 16). Lupus enteritis must be suspected in a young patient with evidence of serositis (pleural effusion, pericardial effusion, or ascites). Imaging appearance of lupus enteritis can mimic CD, although the presence of ascites is not common in CD and should raise suspicion of serositis. Bowel ischemic changes may present mucosal hypoenhancement and serosal hyperenhancement on MRE suggestive for early to intermediate stage bowel ischemia compared to transmural necrosis seen at late-stage ischemia [21]. Similar imaging features might be reported in antiphospholipid antibody syndrome, which is characterized by thrombosis and/or recurrent early pregnancy loss and is highly associated with SLE. However, abdominal vessel thrombosis can be a key diagnostic clue (Fig. 17).

### Systemic sclerosis (Scleroderma)

Systemic sclerosis is an autoimmune disease leading to fibrosis and disfiguration of the skin, along with dysfunctioning lungs, kidneys, heart, and GI tract [118]. Without histopathological investigation, it is difficult to distinguish between systemic sclerosis-related vasculopathy, which is non-inflammatory, and concurrent vasculitis [105]. However, whether due to vasculopathy or vasculitis, GI manifestations are very common in systemic sclerosis, with involvement of the oral cavity, esophagus, stomach, small and large intestine, liver, and pancreas. In nearly 10% of the patients, GI symptoms are the initial presentation of the systemic disease. The small intestine is the second most frequently involved organ, although the majority of patients with intestinal pathologies remain asymptomatic [119].

**Fig. 18:**
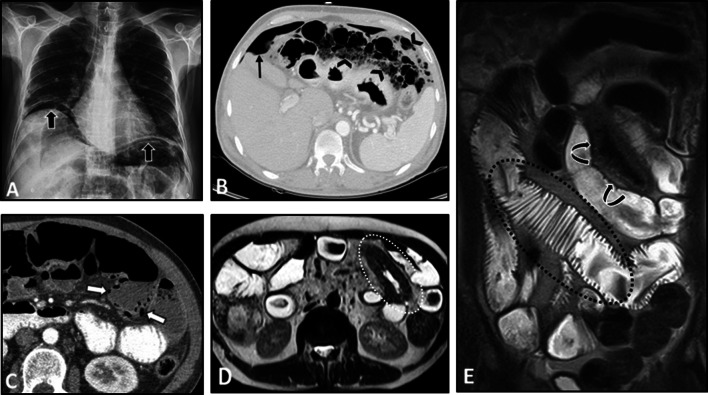
Intestinal vasculitis and benign intestinal pneumatosis in a 52-year-old male with a history of known Scleroderma presenting with abdominal pain. Physical examination revealed generalized abdominal tenderness without rebound or guarding. Upright CXR (**A**) was done in the ER. Pneumoperitoneum (thick black arrows) was noted at CXR in the absence of signs or symptoms of peritonitis. Due to this discrepancy, abdominal CT scan (**B**, **C**) was performed and confirmed pneumoperitoneum (thin black arrow) along with extensive intestinal pneumatosis in the transverse colon (black arrowheads) and jejunum (thick white arrows). The patient underwent conservative treatment. He was discharged one week later with improved signs/symptoms. After three weeks, MRE was obtained and displayed no pneumoperitoneum. Axial T2-W image (**D**) demonstrates segmental jejunal mural thickening (white dotted oval) with hypointense appearance. Coronal T2-W image (**E**) shows bowel dilation and the classic hidebound sign (black dotted oval) in the ileum related to known scleroderma. The T2 hypointense segment of thick jejunum is also seen (curved arrows), suggestive of chronic mural thickening perhaps due to fibrosis

**Fig. 19:**
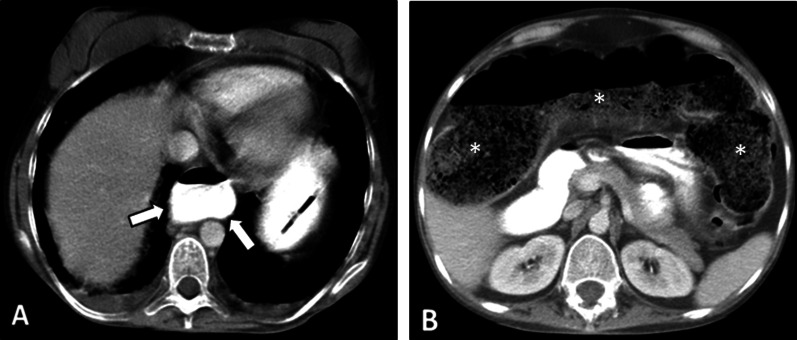
Colonic pseudo-obstruction in a 35-year-old female with known scleroderma presenting with constipation, nausea, vomiting, and abdominal distension. CT scan with IV/oral contrast was done. Axial CT image (**A**) demonstrates dilation of the distal esophagus (thick white arrows), a common finding in scleroderma. Axial CT image (**B**) reveals marked diffuse dilatation of colon (*) without obvious wall thickening or abrupt transition point, suggestive for colonic pseudo-obstruction (Ogilvie syndrome)

Small bowel involvement includes stiffness and atrophy of the smooth muscles leading to reduced small bowel mobility, chronic or acute intestinal pseudo-obstruction, small intestinal bacterial overgrowth, pneumatosis cystoides intestinalis, and jejunal diverticula. Smooth muscle atrophy and fibrosis of the intestinal wall with dominant inner circular muscle layer involvement compared to the outer longitudinal layer result in a characteristic finding in systemic sclerosis, namely “hidebound sign,” which is an increased number of bowel folds stacked together in spite of luminal distention without an increase in interfold distance [120]. Intestinal mural thickening with a hypointense appearance on T2-weighted imaging indicates mural fibrosis (Fig. 18). Additionally, asymmetric small bowel wall fibrosis can result in intestinal sacculation, which may present as multiple wide-mouthed outpouchings involving all intestinal wall layers [120]. Diffuse dilation of the small intestine (particularly jejunum) may occur, mainly upstream to the fibrotic bowel strictures. Colonic dilation may develop following acute colonic pseudo-obstruction, also known as Ogilvie syndrome (Fig. 19). Moreover, pneumatosis cystoides intestinalis, defined as air-filled cysts within the intestinal wall, can be detected on enterography. Rupture of these intraluminal cysts can result in benign or sterile pneumoperitoneum.

**Fig. 20:**
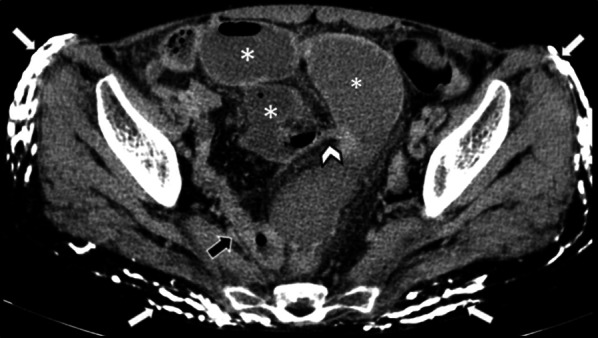
Small bowel obstruction in a 31-year-old female with known scleroderma presenting with generalized abdominal pain, nausea, and vomiting without previous Hx of surgery. Axial view of CTE shows extensive cutaneous and subcutaneous calcifications (thick white arrows). Short segment benign-looking stricture (white arrowhead) is seen at the distal ileum in the pelvis, leading to upstream small bowel dilatation (white asterisks). Following segmental resection and anastomosis, this was confirmed to be a fibrotic stricture. Collapsed ileum distal to stricture is also depicted (thick black arrow)

Subcutaneous calcification is a valuable extra-intestinal finding suggestive of an underlying systemic cause (e.g., scleroderma) for intestinal disease (Fig. 20). Meanwhile, esophageal dilatation and rarely hepatobiliary pathologies (nearly in 1.5% of patients), including hepatic duct obstruction due to vasculitis and as manifestations of primary biliary cirrhosis, can be detected as extra-intestinal pathologies [119].

### Rheumatoid arthritis

Rheumatoid arthritis-associated vasculitis is extremely rare and can occur in patients with long-standing erosive rheumatoid arthritis [107]. Small- and medium-sized vessels are usually involved. GI manifestations may develop in 10–38% of patients with rheumatoid arthritis-associated vasculitis. These presentations include segmental or extensive intestinal infarction, ileal stricture [121, 122], ischemic ulcers of the intestine, and bowel perforation. Rarely other extra-intestinal findings, including hepatomegaly, intrahepatic hemorrhage, and pancreatic necrosis, can be evident [123].

### Single-organ vasculitis (SOV)

This condition rarely occurs in the abdomen, and involvement of the abdominal organs is commonly part of systemic vasculitis. However, SOV in the GI tract has been reported in the stomach, small and large intestine, and most commonly appendix. Abdominal pain is the most frequent manifestation. Bowel infarction, perforation, bleeding, and solid-organ infarction are serious complications of GI SOV [124, 125]. The imaging findings include abnormalities of the corresponding arteries, intestinal wall thickening, bowel infarction, and infarcted areas within solid organs, including the spleen and liver [124].

## Frequent mimickers of vasculitis on MRE/CTE

### Inflammatory bowel disease (IBD)

**Fig. 21:**
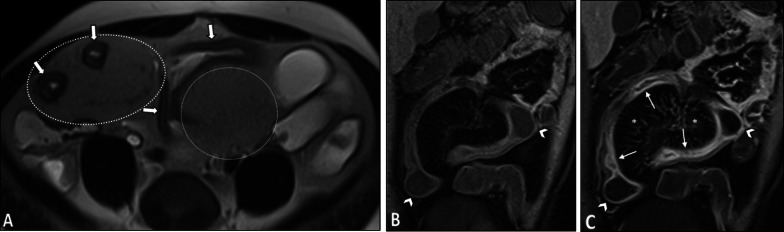
Crohn’s disease in a 23-year-old male presenting with abdominal pain and transient obstructive episodes. MRE was obtained. Axial T2-W image (**A**) shows multi-focal mural thickening in several segments of the ileum (thick white arrows) associated with increased mesenteric fat (white dotted ovals) separating the bowel loops. Coronal pre- and post-contrast T1-W images (**B**, **C**) demonstrate increased striated mural enhancement of the affected bowel segments (thin white arrows), more predominant on the mesenteric border. Engorgement of mesenteric vessels is depicted (white asterisks) representing comb sign. There is also pseudodiverticula formation along the antimesenteric border (white arrowheads)

IBD, particularly CD, is one of the mimickers of AAV or Behçet's disease. The common findings on MRE/CTE include multi-focal intestinal mural thickening, striated mural hyperenhancement, and engorgement of mesenteric vessels. The predominant involvement of the bowel mesentery in CD compared to vasculitis can be an essential discriminating factor. Other helpful clues suggestive of CD are the fibrofatty proliferation of the mesentery and pseudodiverticula formation (Fig. 21). Notably, IBD can occasionally be associated with vasculitis [126]. Therefore, in addition to the clinical presentation, endoscopic and histologic investigations may become necessary to differentiate these entities [127].

### Eosinophilic enteritis

There is an important controversy as to whether eosinophilic gastroenteritis is categorized as the pathogenic spectrum of hypereosinophilic syndrome or EGPA [128]. Imaging findings of eosinophilic enteritis are non-specific, with mural thickening being a common finding. Other radiologic presentations include nodularity, luminal narrowing, and inflammation in the adjacent mesentery [129]. The previous history of allergy and presence of eosinophilia are important diagnostic clues for eosinophilic enteritis. Similar to vasculitis, corticosteroid therapy results in dramatic alleviation of the signs and symptoms [130].

### Graft-versus-host disease (GVHD)

GVHD is a life-threatening complication of allogeneic stem cell or bone marrow transplant (HSCT) involving multiple organ systems, including the GI tract, and frequently is associated with skin involvement. Imaging findings are commonly observed in the GI tract and hepatobiliary system and are non-specific. These include ascites, periportal edema, intestinal wall thickening, mucosal hyperenhancement, bowel dilatation, and submucosal edema, leading to a tubular featureless appearance of the small intestine, called the ribbon sign [131–133]. The main differential diagnoses include neutropenic enterocolitis, infectious enterocolitis, or radiation enteritis. [134]. The location and extent of bowel involvement, clinical history, laboratory findings, and stool studies may aid in distinguishing GVHD from intestinal vasculitis [132].

### Segmental arterial mediolysis (SAM)

**Fig. 22:**
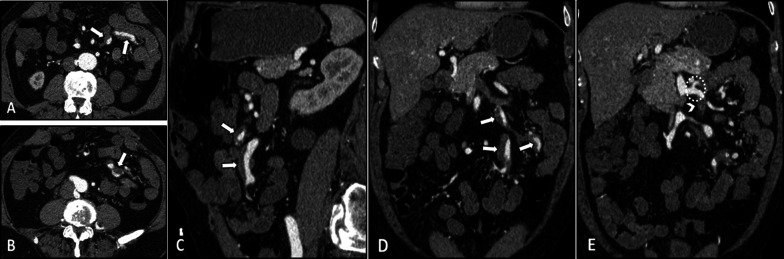
Segmental arterial mediolysis (SAM) in a 55-year-old male presenting with epigastric pain for ten days and normal laboratory results. Axial, sagittal, and coronal images from CTE (**A**–**D**) show fusiform aneurysmal dilatation of two jejunal branches of SMA associated with partial mural thrombosis (thick white arrows). Coronal CTE (**E**) demonstrates aneurysmal dilation and high-grade thrombosis at a jejunal branch of SMA (white arrowhead) with sparing of proximal arterial bifurcation (white dotted oval)

SAM is a non-atherosclerotic and non-inflammatory arteriopathy more commonly affecting middle-aged and elderly patients. It results from lysis of the smooth muscle of the vascular outer layer. Imaging findings in SAM are similar to vasculitis and include aneurysmal dilation, dissection, stenosis, occlusion, and mural thrombosis of splanchnic arteries, particularly in SMA. However, SAM typically involves only the splanchnic arteries and spares other vessels like renal arteries and mesenteric arterial bifurcations from damage (Fig. 22) [12, 135]. Normal levels of inflammatory or immune markers can also help to differentiate SAM from vasculitis.

### Infectious enteritis

Enterography is usually not able to detect infectious enteritis at the early stages. However, chronic infectious diseases, including tuberculosis and cytomegalovirus (CMV), are usually noticeable on CTE and MRE. Findings on enterography include segmental circumferential wall thickening, hyperenhancement, mural edema, and enlargement of the mesenteric lymph nodes [120, 136]. Infectious enteritis should always be considered, particularly in patients with immunodeficiency. Clinical history, laboratory data, and a trial of antibiotic therapy are helpful to confirm the diagnosis. Infectious enteritis is always a challenging differential diagnosis for intestinal vasculitis.

### Angioedema

Angioedema is the swelling of the body following protein extravasation due to increased vascular permeability. The most common causes of angioedema include hereditary or acquired deficiency of C1-esterase inhibitor and medications, such as angiotensin-converting enzyme (ACE) inhibitors [137]. Angioedema commonly presents with cutaneous manifestations; however, in some cases, intra-abdominal involvement may occur without superficial manifestations. Patients with GI involvement commonly present with non-specific manifestations, including abdominal pain, nausea, and vomiting; therefore, imaging plays a key role in diagnosis [138]. Findings on CT include circumferential or asymmetric bowel wall thickening, mural stratification or halo sign due to submucosal edema, prominent mesenteric vessels, ascites, and hyperenhancement [137, 139]. Additionally, MR imaging shows submucosal edema. A thorough evaluation of patient and family history and review of medication usage history in addition to the assessment of complement markers can aid in the differentiation of angioedema from vasculitides [137].

## Diagnostic strategy

**Fig. 23:**
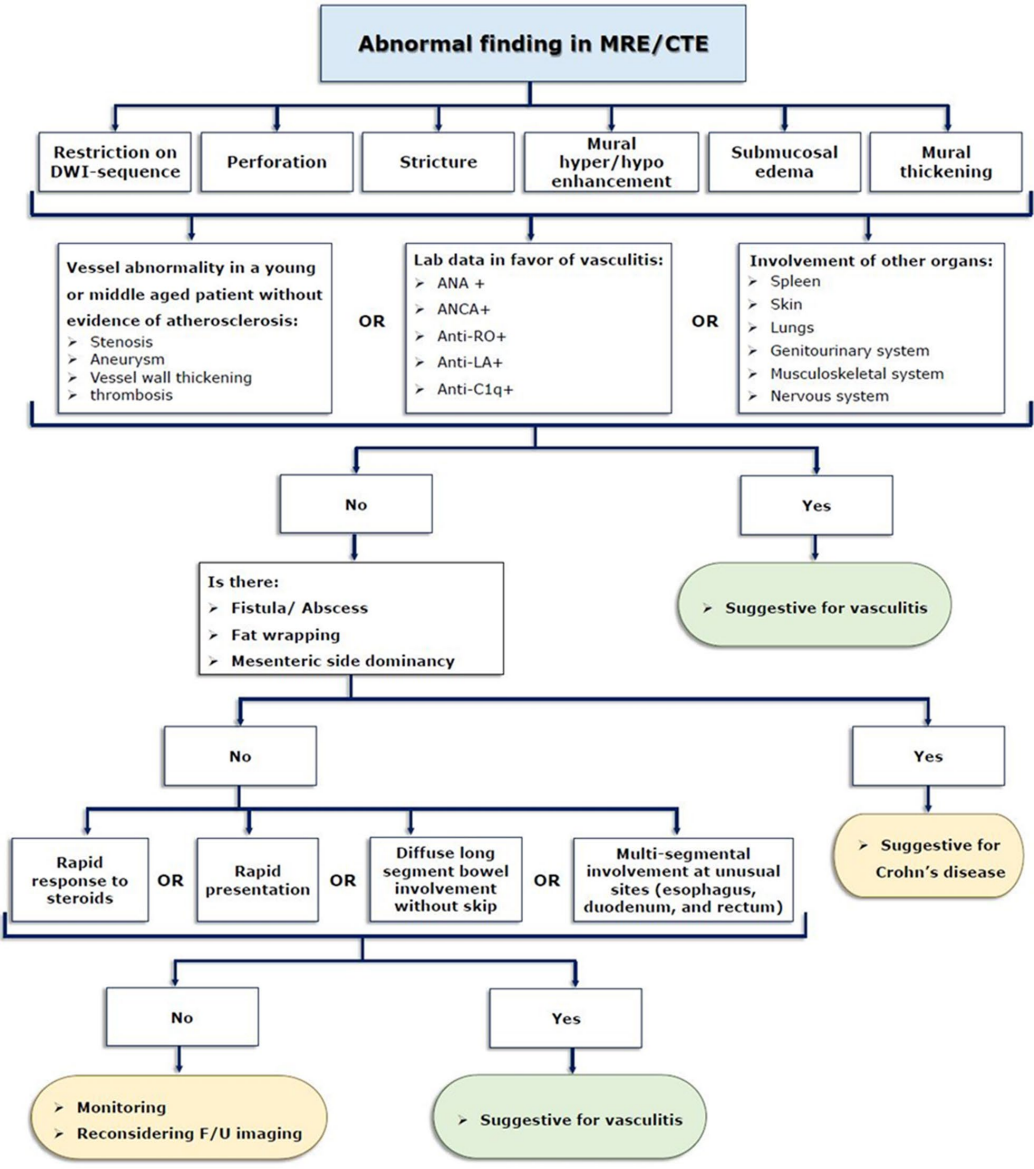
A practical flowchart for potential diagnostic work up of intestinal vasculitis following MRE or CTE

**Fig. 24:**
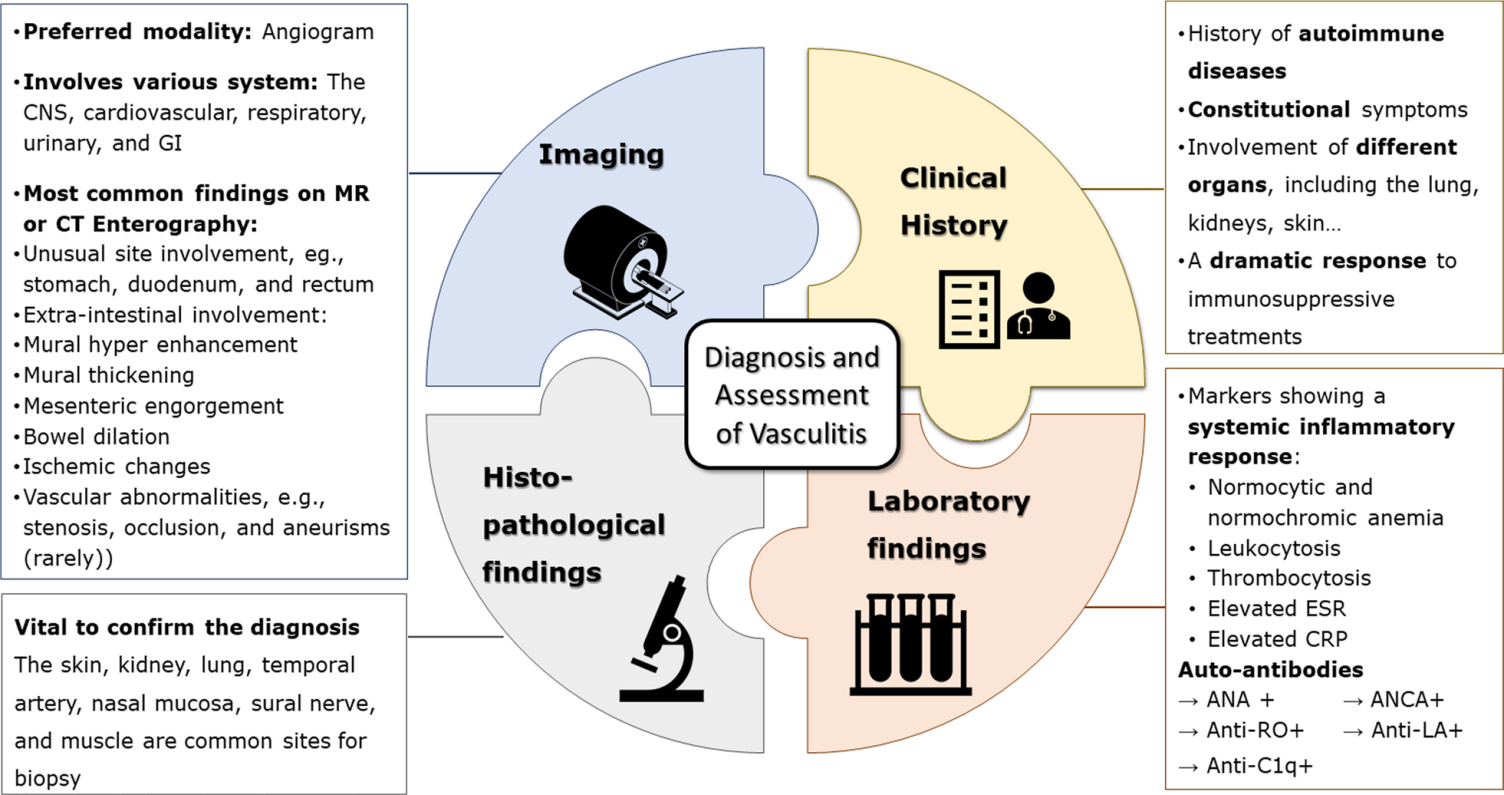
The diagnosis of intestinal vasculitis is based on a combination of the clinical manifestation and history, imaging, laboratory, and histopathology findings

Figure 23 presents a flowchart for inference about a potential diagnosis of intestinal vasculitis on MRE or CTE. Imaging features are often non-specific, including mural thickening, submucosal edema, mural hyper/hypoenhancement, and rarely stricture or perforation. Positive laboratory biomarkers, multi-organ involvement, and the presence of vascular abnormalities, especially in a young/middle-aged patient without evidence of atherosclerosis, raise the clinical suspicion of vasculitis. Since intestinal vasculitis often mimics CD, a careful inspection must be done searching for fistula/abscess, mesenteric fat wrapping, or dominant involvement of the adjacent mesenteric border. Moreover, rapid response to corticosteroids, acute presentation, multi-segmental involvement at unusual sites, i.e., esophagus, duodenum, and rectum, or diffuse long segmental bowel involvement without skip may also be suggestive of vasculitis. Infectious enteritis is another differential, particularly in immunocompromised patients. As a challenging issue, any suspicion of infectious enteritis may postpone life-saving treatment with corticosteroid or immunosuppressive agents in patients with vasculitis. Diagnosis of intestinal vasculitis (Fig. 24) relies on a combination of history, clinical presentation, imaging, laboratory findings, and histopathology. The radiologists need to be aware of the findings suggestive of the diagnosis of intestinal vasculitis to avoid potential missed or late diagnosis. Table 1 summarizes clinical clues and enterography findings of common vasculitides involving the GI tract.

**Table 1:** Vasculitides with detected complications on enterography

Vasculitis	Clinical clues	Imaging pearl for the radiologists	Mimickers on MRE/CTE
Large-vessel vasculitis			
Takayasu arteritis	Age at onset ≤ 40 y Claudication of the extremities Decreased brachial artery pulse Blood pressure difference > 10 mm Hg between the arms Bruit over the subclavian arteries or aorta Abdominal bruit in 14%	Involvement of the aorta and the origin of major visceral arteries, presenting with concentric mural thickening, transmural calcification, luminal stenosis, occlusion, aneurysmal changes, and collateral vessels formation Double ring vascular enhancement pattern Non-specific intestinal mural thickening, submucosal edema, and enhancement	Mesenteric ischemia due to other etiologies Atherosclerosis Connective tissue disorders Marfan syndrome Ehlers-Danlos syndrome Loeys-Dietz syndrome
Giant cell arteritis	Patient age > 50 years New-onset headache Temporal artery abnormality (tenderness or decreased pulsation) ESR ≥ 50 mm/h Abnormal temporal artery biopsy results Abdominal pain may indicate aorta aneurysm or dilation	Signs of mesenteric ischemia	
Medium-vessel vasculitis			
Polyarteritis nodosa	Disease-associated weight loss ≥ 4 kg Livedo reticularis Testicular pain or tenderness Myalgia, weakness, or leg tenderness Mono- or polyneuropathy Diastolic blood pressure > 90 mm Hg Elevated serum levels of creatinine or blood urea nitrogen Presence of hepatitis B reactants in serum Biopsy of a small- or medium-sized artery containing neutrophils or mixed leukocyte infiltrate	Involvement of mesenteric and visceral arteries, e.g., stenosis, vessel irregularity, aneurysmal dilation, and arterial mural thickening, with a predilection for superior mesenteric artery (SMA) branches Segmental bowel mural thickening Submucosal edema and mural hyperenhancement with a striated pattern Visceral infarction (liver, spleen, kidneys, and intestine) Spontaneous abdominal hemorrhage secondary to a ruptured aneurysm Heterogeneous liver or renal enhancement due to microvascular abnormalities	Segmental arterial mediolysis (SAM) Fibromuscular dysplasia (FMD) Mycotic aneurysm
Small-vessel vasculitis			
*ANCA- associated vasculitis*			
Granulomatosis with polyangiitis (Wegener's granulomatosis)	Nasal and oral inflammation: oral ulcers or bloody nasal discharge Microhematuria Granulomatous inflammation of arterial walls or extravascular tissue	Intestinal ischemia presenting with multi-focal segmental circumferential mural thickening and abnormal hyperenhancement Abnormal chest radiographic findings: multiple and bilateral pulmonary nodules fixed infiltrates, or masses, or alveolar hemorrhage Renal pathologies, i.e., ischemia and interstitial nephritis Visceral infarction (liver, spleen, and kidneys)	Non-occlusive mesenteric ischemia Infectious enteritis Eosinophilic enteritis Inflammatory bowel disease Radiation and chemotherapy induced enteritis
Eosinophilic granulomatosis with polyangiitis (Churg-Strauss)	Asthma Eosinophilia > 10% Mono- to polyneuropathy Nonfixed pulmonary infiltrates Paranasal sinus abnormality Extravascular eosinophils	Submucosal edema and mural hyperenhancement with a striated pattern with or without thickening (halo sign) Bowel dilatation, stenosis, or obstruction Hepatobiliary complications, i.e., cholestasis and liver infarction Pulmonary involvement (Non-Fixed parenchymal opacities)	
Microscopic polyangiitis	Rapid progressive glomerulonephritis and/or alveolar hemorrhages Histopathologic findings of small vessel vasculitis or necrotizing glomerulonephritis Symptoms suggestive of small vessel involvement Skin lesions (commonly presented as palpable purpura) Neurological manifestations	Concentric mural thickening Post-contrast T1-weighted hyperenhancement Engorgement of mesenteric vessels Signs of bowel infarction or perforation Renal pathologies (striated pattern) Pulmonary pathologies	
*Immune complex-associated vasculitis*			
IgA vasculitis	Age ≤ 20 years at disease onset Palpable purpura Acute abdominal pain Biopsy showing granulocytes in the walls of small vessels (presence of 2 or more) Arthralgia and/or arthritis Glomerulonephritis	Signs of bowel ischemia and edema, e.g., segmental mural thickening and post-contrast focal mural hypo-enhancement Self-limiting mucosal and submucosal hemorrhage Signs of bowel perforation, obstruction, or irreducible intussusception Gallbladder wall thickening	
Variable-vessel vasculitis			
Behçet disease	*Mandatory criteria* Recurrent oral aphthosis *Minor criteria* Ocular lesions Recurrent genital aphthosis Skin lesions Positive pathergy test	Predominant ileocecal involvement Irregular circumferential mural thickening with homogeneous mural enhancement Deep penetrating ulcers and restricted diffusion on DWI in the involved section No specific predilection to the mesenteric side No surrounding mesenteric inflammatory changes	Non-occlusive mesenteric ischemia Infectious enteritis Eosinophilic enteritis Inflammatory bowel disease Radiation and chemotherapy-induced enteritis
Vasculitis associated with systemic diseases			
Systemic Lupus Erythematosus (SLE)	Synovitis or tenderness in at least two joints Cutaneous presentations, e.g., malar rash, discoid rash, and photosensitivity Oral ulcers Hematologic abnormalities, including thrombocytopenia, autoimmune hemolysis, and leukopenia Neurological involvement, such as seizure, delirium, or psychosis Proteinuria or lupus nephritis Low C3 and/or C4 Anti dsDNA antibody and/or Anti-Smith antibody positive	Multi-focal bowel wall thickening not confined to a single vascular territory Submucosal edema and mural hyper enhancement Target sign (diffuse circumferential wall thickening with submucosal edema) Evidence of serositis, e.g., ascites Comb sign (The hypervascular appearance of the mesentery) Genitourinary involvement, e.g., hydronephrosis, cystitis, and lupus nephritis Signs of solid-organ infarction, including wedge-shaped renal and splenic infarcts	Non-occlusive mesenteric ischemia Infectious enteritis Eosinophilic enteritis Inflammatory bowel disease Radiation and chemotherapy-induced enteritis
Systemic sclerosis	Skin thickening of the fingers Fingertip lesions Telangiectasia Abnormal nailfold capillaries Pulmonary arterial hypertension and/or Interstitial lung Disease Raynaud's phenomenon anti-centromere and/or anti-topoisomerase antibody positive	Benign or sterile pneumatosis Hidebound sign (increased number of bowel folds stacked together despite luminal distention without an increase in interfold distance) Diffuse dilation of the small intestine (particularly jejunum) Intestinal mural thickening, indicating mural fibrosis Multiple wide-mouthed outpouchings involving all intestinal wall layers Subcutaneous calcifications	

## Limitations

While this review provides a comprehensive summary of imaging findings of intestinal involvement in vasculitis with the demonstrative cases, it faces several limitations. The sparse data on MRE and CTE findings of vasculitis were the major limitation of the literature, which might have affected this study. We did not identify any report in the literature about the MRE and CTE of some vasculitides, such as Takayasu arteritis, GCA, Kawasaki disease, and HSP. This review highlighted this gap in the literature warranting further investigation. Moreover, we could not include the images from a limited number of cases such as GCA, Kawasaki disease, and cryoglobulinemic vasculitis due to the very low frequency of these cases with intestinal involvement.

## Conclusion

While gastrointestinal presentations are uncommon manifestations in vasculitis, their timely diagnosis holds immense importance. Establishing the diagnosis is hindered by the heterogeneous and non-specific manifestations of the vasculitides and usually requires a combination of clinical, imaging, laboratory, and histopathological data. MRE and CTE may be utilized in diagnostic investigations of patients presenting with GI involvement of a previously undiagnosed or diagnosed vasculitis. As previously described in detail, several key technical points on MRE/CTE protocols should be considered in these patients. In order to make the correct diagnosis, the imaging features need to be linked with the relevant clinical and laboratory data in a two-way street.

## Data Availability

Data sharing is not applicable to this article as no datasets were generated or analyzed during the current study.

## Abbreviations

AAV: Antineutrophil cytoplasmic antibody (ANCA)-associated vasculitis
ACE: Angiotensin-converting enzyme
ANCA: Antineutrophil cytoplasmic antibody
CD: Crohn's disease
CMV: Cytomegalovirus
CRP: C-reactive protein
CTE: Computed tomography enterography
EPGA: Eosinophilic granulomatosis with polyangiitis
ESGAR: European Society of Gastrointestinal and Abdominal Radiology
ESPR: European Society of Pediatric Radiology
ESR: Erythrocyte sedimentation rate
FMD: Fibromuscular dysplasia
GCA: Giant cell arteritis
GI: Gastrointestinal
GPA: Granulomatosis with polyangiitis
GVHD: Graft-versus-host disease
HSP: Henoch–Schönlein purpura
IBD: Inflammatory bowel disease
Ig: Immunoglobulin
MPA: Microscopic polyangiitis
MRE: Magnetic resonance enterography
PAN: Polyarteritis nodosa
PET: Positron emission tomography
SAM: Segmental arterial mediolysis
SLE: Systemic lupus erythematosus
SMA: Superior mesenteric artery
SOV: Single-organ vasculitis
